# Comparative Gas-Sensing Studies of EPAA with Aniline Tetramer in the Main/Side Chain and Corresponding Densely/Porous Electro-Spun Films

**DOI:** 10.3390/polym18202457

**Published:** 2026-10-09

**Authors:** Kung-Chin Chang, Ting-Ying Lai, Kun-Hao Luo, Yung-Chen Lin, Yen-Lin Lin, Jui-Ming Yeh

**Affiliations:** 1 Department of Textile Technology and Innovation Application Engineering, Chinese Culture University, Yang-Ming-Shan, Taipei City 11114, Taiwan; 2 Department of Chemistry, Chung Yuan Christian University, Chung Li District, Taoyuan City 32023, Taiwan; betty901003@gmail.com (T.-Y.L.); khluo1997@gmail.com (K.-H.L.); jacky29612000@gmail.com (Y.-C.L.); gary07182003@gmail.com (Y.-L.L.)

**Keywords:** branched-type, electroactive poly(amic acid), electrospinning, H_2_S gas-sensing

## Abstract

Rapid low-ppm detection of toxic and corrosive hydrogen sulfide (H_2_S) is essential to protect workers and the public. This research presents the synthesis and gas-sensing application of two electroactive poly(amic acid)s (EPAAs): a linear-type (L-EPAA) and a branched-type (B-EPAA). Synthesized via condensation polymerization using electroactive diamine monomers composed of linear and pendant side-chain double-terminal aniline tetramers, their chemical structures, molecular weight, electroactivity, and conductivity were verified using ATR-FTIR, GPC, cyclic voltammetry, and four-point probe testing. To significantly increase the specific surface area, electrospinning technology was employed to fabricate a porous fiber film (B-EPAA-ES). All samples were deposited onto interdigitated electrodes to evaluate their H_2_S gas sensing properties. Among the tested results, the electrospun B-EPAA-ES film demonstrated the most outstanding performance, exhibiting a 47.4% response to 10 ppm of H_2_S with a rapid response time of 53.1 s. Moreover, the sensor displayed remarkable reproducibility (CV of 0.2% over three cycles) and the H_2_S response is unaffected by other gases. Ultimately, the B-EPAA-ES film combines high processability of poly(amic acid), reversible redox properties of side-chain aniline tetramers, and a large specific surface area. This synergistic combination enables highly repeatable H_2_S detection and the response is unaffected by other gases.

## 1. Introduction

Hydrogen sulfide (H_2_S) is a colorless, highly toxic, and combustible gas generated through natural decomposition and various industrial operations, including kraft pulping mills, landfills, coke manufacturing, and oil and gas extraction and refining, as well as wastewater management. The health hazards associated with H_2_S depend heavily on its concentration levels. Even minimal exposure measured in low parts-per-million (ppm) can lead to severe consequences: approximately 2 ppm can trigger nausea, 20 ppm can cause a loss of smell, 100 ppm leads to acute respiratory irritation, and exposure exceeding 250 ppm can be lethal [1]. Consequently, maintaining continuous, real-time monitoring of H_2_S at sub-ppm concentrations is essential for safeguarding both occupational health and the environment.

A wide range of organic and inorganic semiconducting materials have been explored to improve hydrogen sulfide (H_2_S) sensors, including metal-oxide-semiconductor [2,3], electrochemical [4,5], organic [6], and liquid crystal (LC) variants [7,8,9]. For example, Chen et al. investigated the development of high-performance H_2_S gas sensors using both unpolymerized and UV-polymerized thin films based on a polymerizable discotic liquid crystal (DLC) derivative. Operating via a protic acid doping mechanism under humid conditions, these sensors demonstrate high selectivity, a low detection limit of 1 ppm, and stable performance for up to 30 days [9]. Yang et al. introduces a Pt/CrWN_2_-based electrochemical gas sensor designed for fast, reliable, and cost-effective real-time detection of H_2_S in wastewater. The sensor exhibits a high sensitivity of 0.295 μA/ppm and rapid response and recovery times of 3.6 and 3.5 s and remains stable for up to 90 days even after direct sewage exposure [10]. Tai et al. presented a highly uniform Pd/WO_3_ H_2_S sensor fabricated via sequential e-beam evaporation. Operating at an optimal temperature of 250 °C, the sensor exhibits outstanding gas-sensing properties, including a high sensitivity of 2.68 ppm^−1^, a short response time of 14 s, and an ultra-low detection limit of 50 ppb [11]. Zulfiqar et al. detailed the synthesis of pure and Fe-doped SnO_2_ nanoparticles using a co-precipitation method to develop high-performance gas sensors. Among the synthesized samples, the 3 wt% Fe-doped SnO_2_ sensor demonstrated an exceptional gas response of 92.028 to 100 ppm of H_2_S gas at an optimal operating temperature of 275 °C, showing outstanding selectivity over other reducing gases such as NH_3_, CO, and C_3_H_6_ [12]. 

While inorganic materials demonstrate excellent H_2_S detection capabilities, they typically require high operating temperatures between 75 °C and 400 °C. This heat requirement makes them impractical for room-temperature environments where safeguarding humans from toxic H_2_S exposure is crucial. Consequently, conjugated polymers have recently gained significant traction as active sensing materials. This growing interest is driven by their lightweight and flexible nature, ease of chemical modification to enhance device sensitivity and selectivity, compatibility with solution processing, and promise for cost-effective manufacturing [13,14,15].

Many studies have been conducted on introducing aniline oligomers (e.g., dimers, trimers, and tetramers) into polymer matrices to form electroactive polymers [16,17,18]. Some of these studies have investigated their gas sensing performance [19,20,21,22,23,24]. Besides using electroactive polymers with conjugated structures, the most effective way to improve gas sensing performance is to increase the surface area of the material, including methods such as biomimetic structures [25,26], supercritical fluid technology [27,28], and electrospinning technology [29,30,31], etc.

To the best of our knowledge, previous research has investigated the gas sensing properties of electroactive polymers, including those incorporating electroactive aniline oligomers into various polymer matrices such as polyimide (PI) [20], polylactic acid (PLA) [21], polyurethane-urea (PUU) [22], polymethacrylate (PMMA) [23], and poly(amic acid) (PAA) [24], the application of electroactive polymers combined with electrospinning technology for gas sensing remains largely unexplored [30]. In view of this, this study is the first to examine the influence of electroactive aniline oligomers into the main backbone or side chain of the EPAA structure on H_2_S gas sensing. Furthermore, this study explores in more detail the EPAA porous fiber films with different fiber diameters prepared under various electrospinning conditions. Since electrospinning technology can increase the specific surface area of materials, it allows for a more in-depth investigation of its gas sensing capabilities.

## 2. Materials and Methods

### 2.1. Chemicals and Materials

N-phenyl-1,4-phenylene diamine (99%; Sigma-Aldrich, St. Louis, MO, USA), 4,4-diaminodiphenylamine sulfate hydrate (97%; Sigma-Aldrich), ammonium peroxodisulfate (APS, 98%; Merck, Darmstadt, Germany), N,N-dimethyl formamide (DMF, 99%; Merck), triethylamine (TEA, 99%; J.T. Baker, Phillipsburg, NJ, USA), dichloromethane (DCM, 99.5%; Macron, Crespellano, Italy), N,N-dimethylacetamide (DMAc, 99%; Macron), 2,6-difluorobenzoyl chloride (DFB, 98%; Alfa Aesar, Ward Hill, MA, USA), 4-aminophenol (99%; Sigma-Aldrich), toluene (99.5%; J.T. Baker), hydrochloric acid (HCl, 37%; Riedel-deHaën, Seelze, Germany), ammonium hydroxide solution (NH_4_OH, 25%; Riedel-deHaën), 4,4′-oxydianiline (ODA; 97%; Sigma-Aldrich), and 1,2,4,5-pyromellitic dianhydride (PMDA; 97%) were used as received without further purification. All gases used in this study, including dry air, hydrogen sulfide (H_2_S), nitrogen dioxide (NO_2_), carbon monoxide (CO), and sulfur dioxide (SO_2_), were at a concentration of 50 ppm (Chian Hong Gas Co., Ltd., Hong Kong, China) and used as received. Indium tin oxide glass (ITO, Ruilong Optoelectronics, Miaoli County, Taiwan) was cut into a plate measuring 25 mm × 25 mm in size and having a thickness of 0.75 mm.

### 2.2. Instruments

A Fourier-transform infrared (FTIR) spectrometer (JASCO, FT/IR-4200, Tokyo, Japan) was employed to acquire the FTIR spectra for wavelengths ranging from 4000 to 400 cm^−1^. Mass spectra were obtained using a Bruker Daltonics IT mass spectrometer model Esquire 2000 (Leipzig, German) with an Agilent ESI source (model G1607-6001). Attenuated total reflectance (ATR), a variant of FTIR spectroscopy, was measured using an FTIR spectrometer (FTIR-4200, JASCO, Oklahoma City, OK, USA; resolution, 4.0 cm^−1^; room temperature, 25 °C ± 0.5 °C) at 4000-400 cm^−1^. Weight-average and number-average molecular weights of EPAA polymers were tested on a Waters GPC-150CV (USA) equipped with a differential refractometer detector and a Styragel HT column using NMP as eluent and monodisperse polystyrene as calibration standards. The electrochemical measurements were conducted using an Autolab workstation (PGSTAT204, Metrohm, Herisau, Switzerland) equipped with a standard three-electrode setup. In this configuration, a coated stainless-steel substrate served as the working electrode, paired with a platinum plate counter electrode and a saturated calomel reference electrode. Electrode potential was examined for up to 10 cycles between −0.2 and 1.0 V to capture the redox transitions [32] and using a 100 mV/s scan rate to provide optimal conditions to fully utilize the electrochemical activity of materials [33]. The morphologies of the materials were determined via scanning electron microscopy (SEM; JEOL JSM-7600F, Tokyo, Japan). The N_2_ adsorption–desorption isotherm (BET, Micromeritics ASAP-2010, GA, USA) was used to determine the surface area and pore diameter of the prepared materials in the relative pressure (P/P0) range of 0–1. The desorption data of N_2_ isotherm were analyzed by the Barrett−Joyner−Halenda (BJH) method to acquire the value of specific surface area and pore size of the prepared materials. For the BET measurement, the weight of each testing sample was about 20 mg. The degassing condition was vacuumed to 10 µmHg, then heated to 80 °C and maintained for approximately 19 h. Following adsorption with N_2_ gas, the prepared samples were tested. Conductivity measurements of the sensors were performed using a four-point probe (LRS4-TK1, KeithLink Technology, Taipei, Taiwan). The H_2_S sensing was performed by testing a self-made gas-sensing system with a Keithley 2450 Source Meter.

### 2.3. Preparation of Electroactive Monomers

#### 2.3.1. Synthesis of Bilaterally Amino-Terminated Aniline Tetramer (BATAT)

**Scheme 1:**
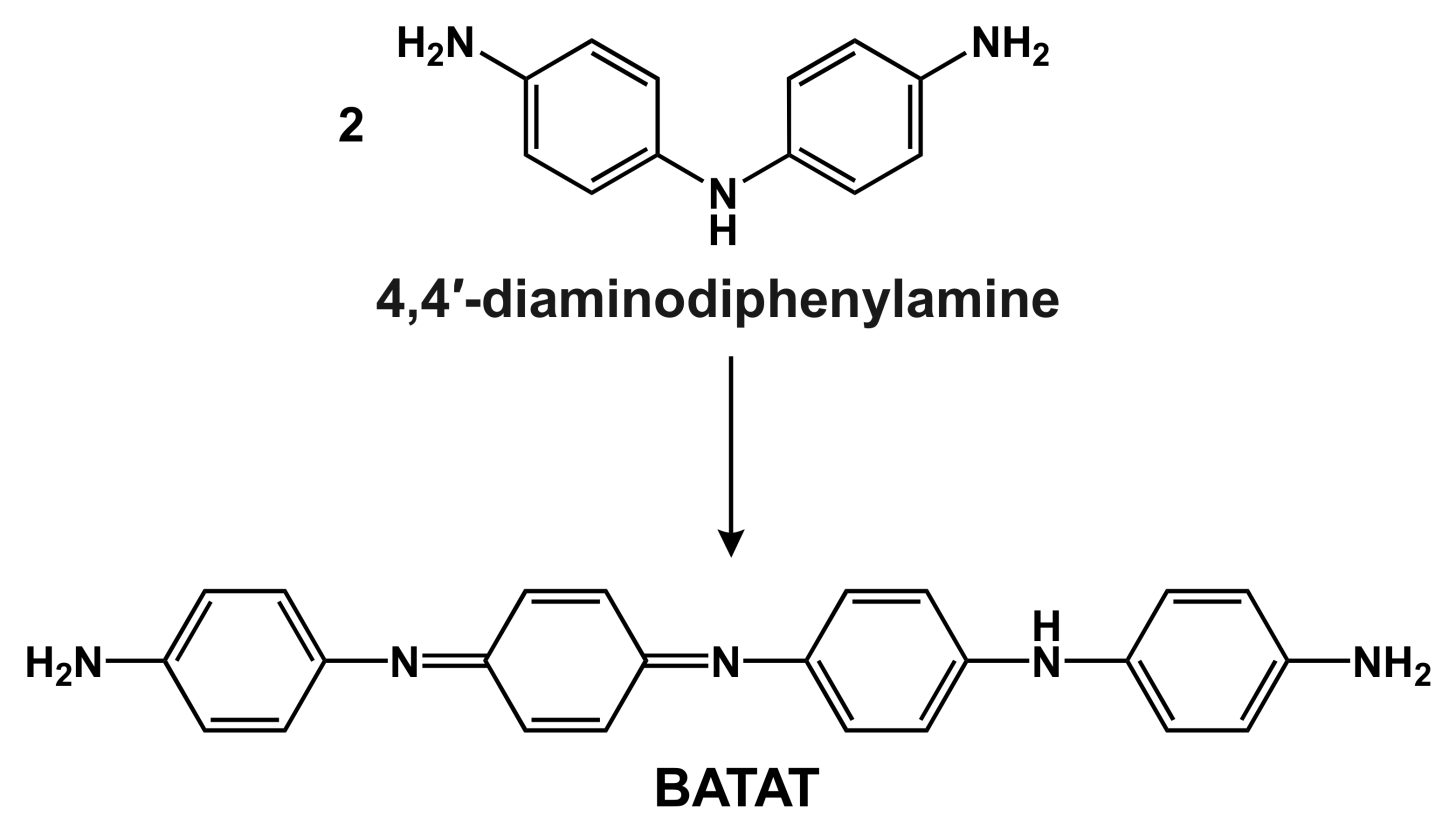
Synthetic route of BATAT.

The synthesis of BATAT was carried out following the procedure previously described in the literature [34], as illustrated in Scheme 1.

#### 2.3.2. Synthesis of Unilaterally Amine-Terminated Aniline Tetramer (UATAT), Aniline Tetramer-Difluorobenzene Monomer (DFAT) and Side Chain Electroactive Diamine Monomers (SCEDA)

**Scheme 2:**
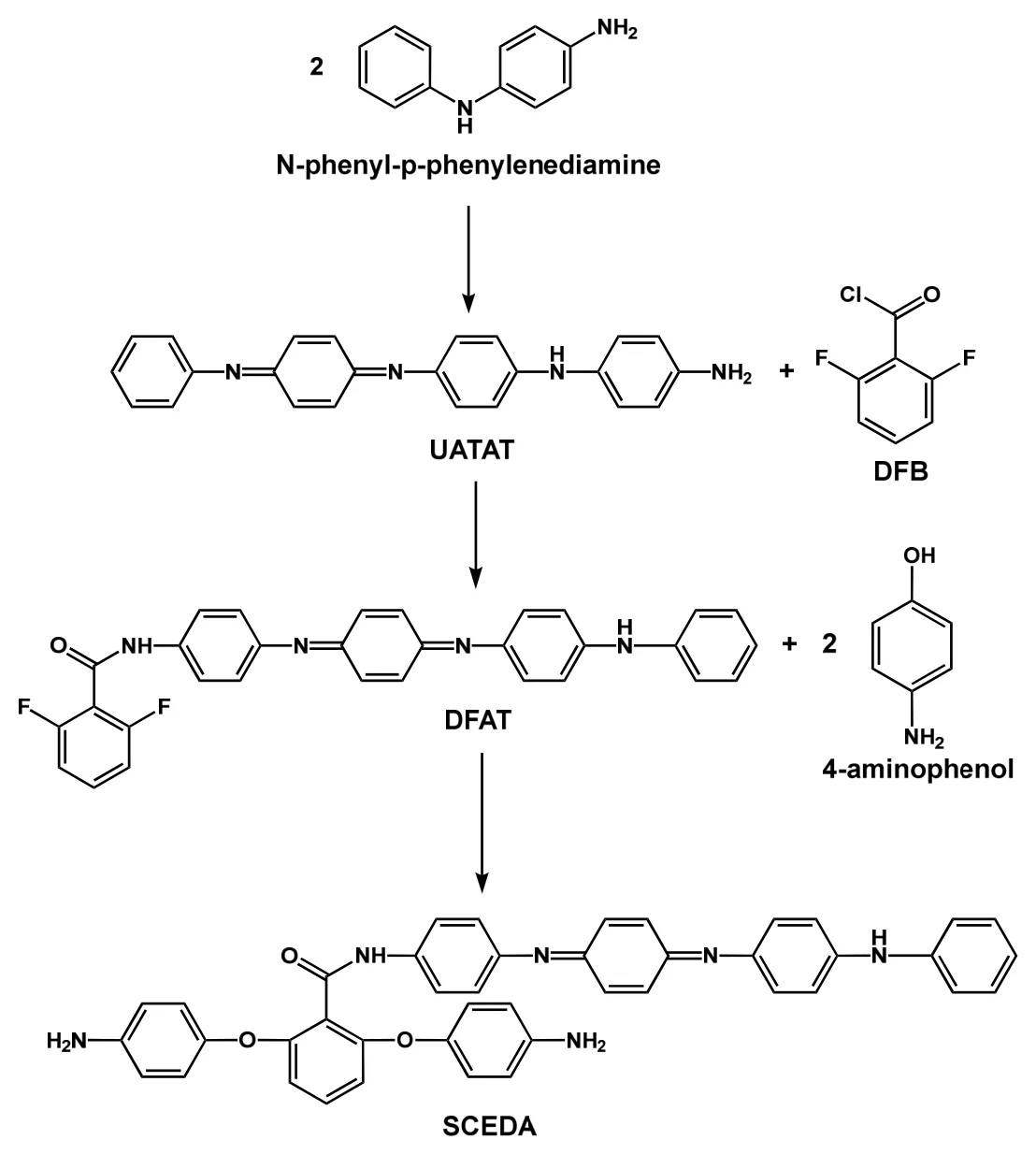
Synthetic route of SCEDA.

UATAT, DFAT, and SCEDA were synthesized by the procedure reported in the literature [24], as shown in Scheme 2.

### 2.4. Synthesis of Main Chain (Linear) Electroactive Poly(amic Acid) (L-EPAA) and Side Chain (Branch) Electroactive Poly(amic Acid) (B-EPAA)

**Scheme 3:**
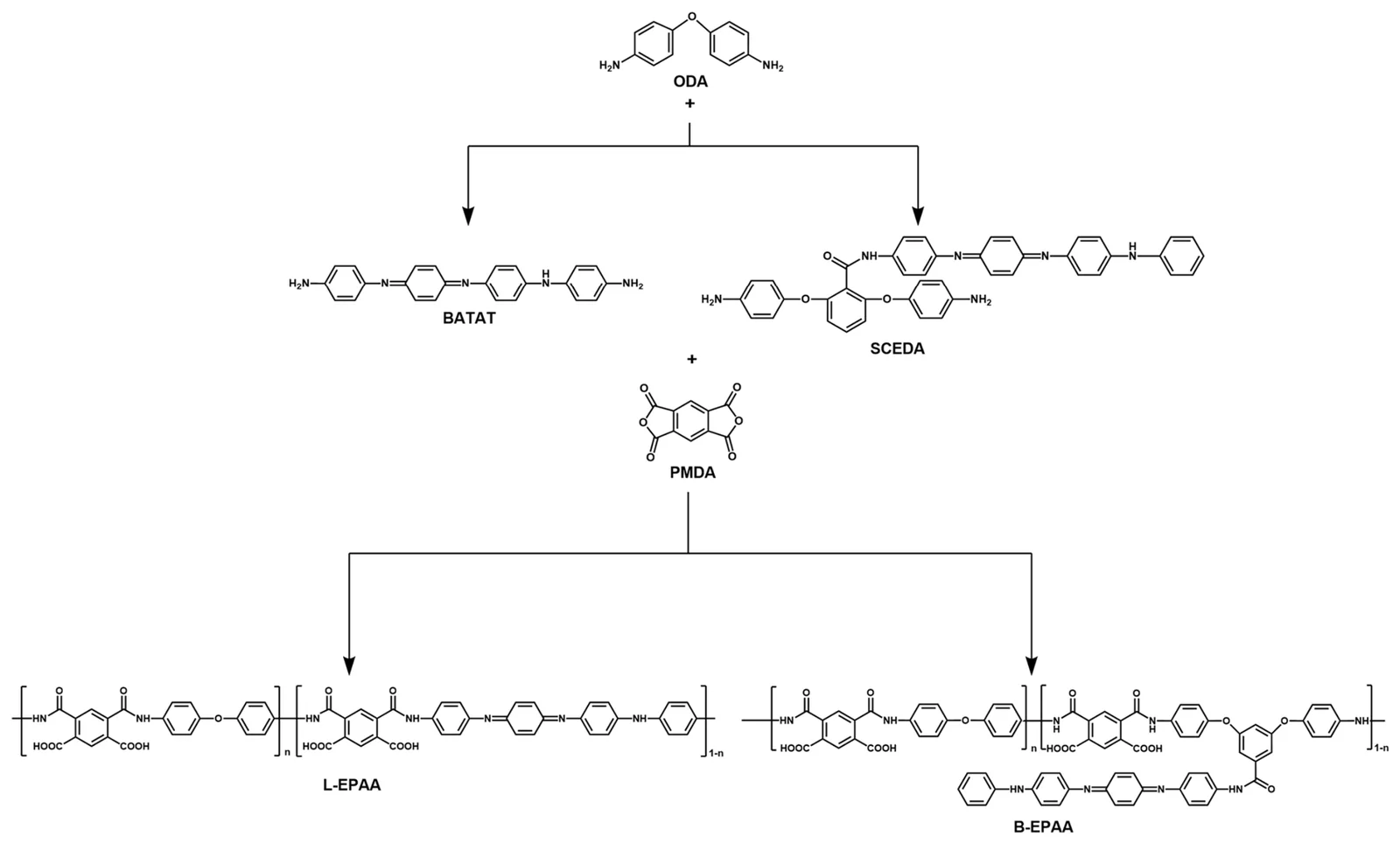
Schematic illustration of the process for the synthesis of L-EPAA and B-EPAA.

Scheme 3 depicts the synthetic pathway for the L-EPAA and B-EPAA. ODA (1.586 g, 8 mmol) and 1 mol % BATAT (0.031 g)/SCEDA (0.055 g) were dissolved in DMF (27.38 mL), respectively, then heated to 50 °C under nitrogen purge throughout and stirred for 30 min until completely dissolved. After that, PMDA (1.763 g, 8.08 mmol) was added in four portions, 15 min apart, and the reaction was carried out for 10 h to obtain the L-EPAA and B-EPAA viscous liquids, respectively.

### 2.5. Preparation of EPAA Films (EPAA-F)

Drops of the L-EPAA and B-EPAA viscous liquids were spread on a Petri dish, respectively. The Petri dish was then placed in an oven at 60 °C for 5 h to completely dry. The L-EPAA and B-EPAA films (L-EPAA-F and B-EPAA-F) were obtained.

### 2.6. Preparation of B-EPAA Porous Fiber Film via Electrospinning (B-EPAA-ES)

**Scheme 4:**
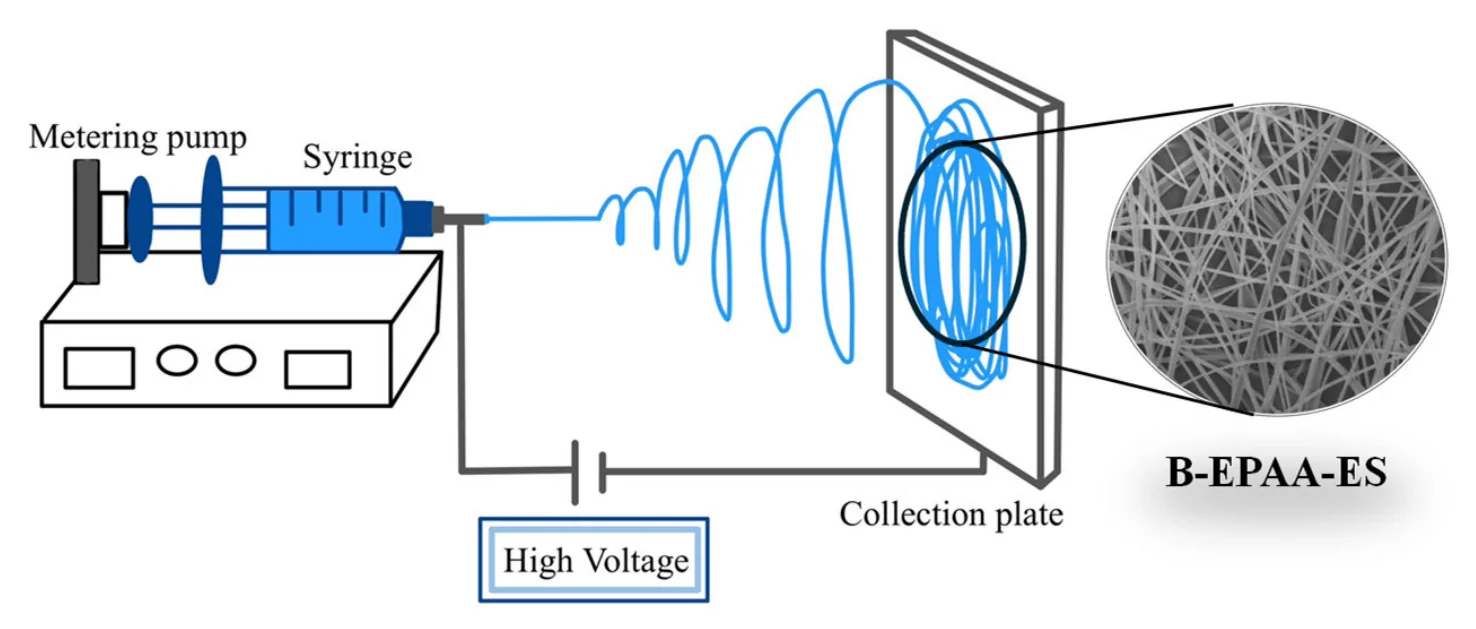
Electrospinning of B-EPAA porous fiber film.

The B-EPAA viscous liquid prepared according to the procedure described above was dissolved in DMF to prepare an electrospinning solution in the viscosity of about 1500 cP. This solution was placed in a 5 mL plastic syringe, and the B-EPAA fiber was fabricated by electrospinning, controlling specific parameters (Q, H, and V). The electrical field used for electrospinning was generated by a variable high-voltage power supply. The solution was fed by a syringe pump, whose feeding rate was tuned for polymer solutions (Q = 0.01, 0.03 mL/min). A metallic needle was connected to ahigh-voltage source set at 13 or 15 kV, and nozzle to-collector distances of 6 and 10 cm were set to collect the electrospun fiber mat samples. The collected mat was then baked in a vacuum oven for 5 h at a temperature of 60 °C to remove residual solvent, resulting B-EPAA porous fiber film, as shown in Scheme 4.

### 2.7. Fabrication of L-EPAA-F-, B-EPAA-F-, and B-EPAA-ES-Coated Gas Sensor

**Scheme 5:**
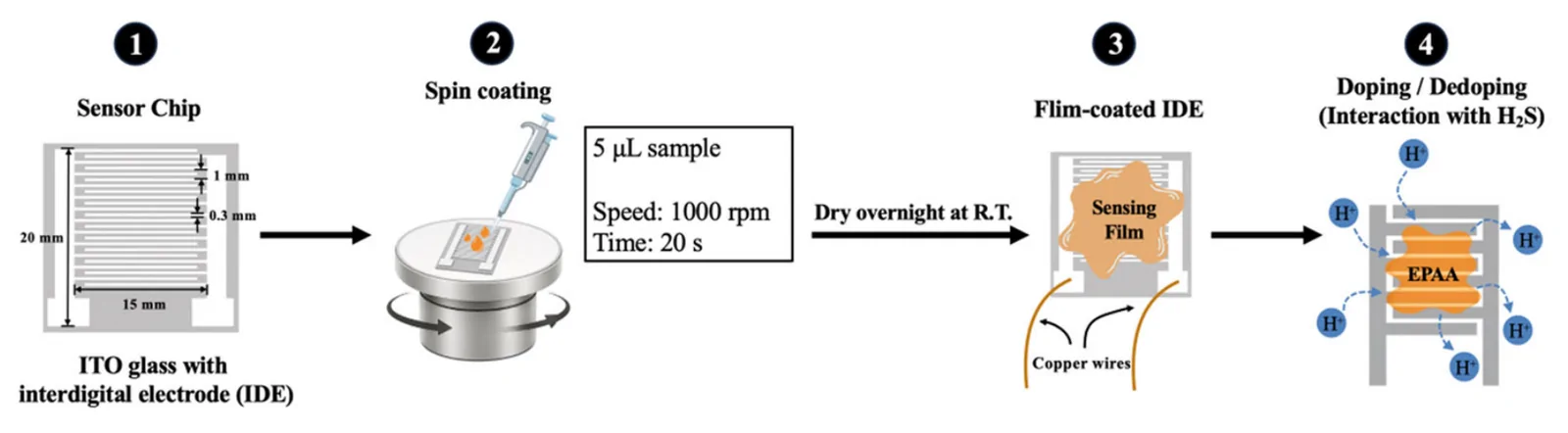
Fabrication of L-EPAA-F- and B-EPAA-F-coated sensors.

L-EPAA-F and B-EPAA-F were coated on indium tin oxide (ITO) substrates by spin-coating, and B-EPAA-ES was fabricated on ITO substrates by electrospinning. For L-EPAA-F and B-EPAA-F, initially, 0.05 wt% solutions of the two samples were mixed in DMF with continuous stirring under ambient conditions (Scheme 5). To form the active sensing layers, 5 µL aliquots of the respective mixtures were dropped onto the ITO surfaces and spun at a speed of 1000 rpm and time of 20 s. Finally, the deposited layers were left to dry overnight at room temperature (25 ± 0.5 °C), successfully forming homogeneous coatings with a thickness measuring approximately 100 ± 10 nm [24]. For B-EPAA-ES, the sensing layer fabrication on ITO was carried out following the procedure of Section 2.6. The parameters of Q, H, and V were 0.03 mL min^−1^, 6 cm, and 13 kV. The thickness of porous fiber film was approximately 130 ± 20 nm.

### 2.8. Gas Sensing Measurements

**Scheme 6:**
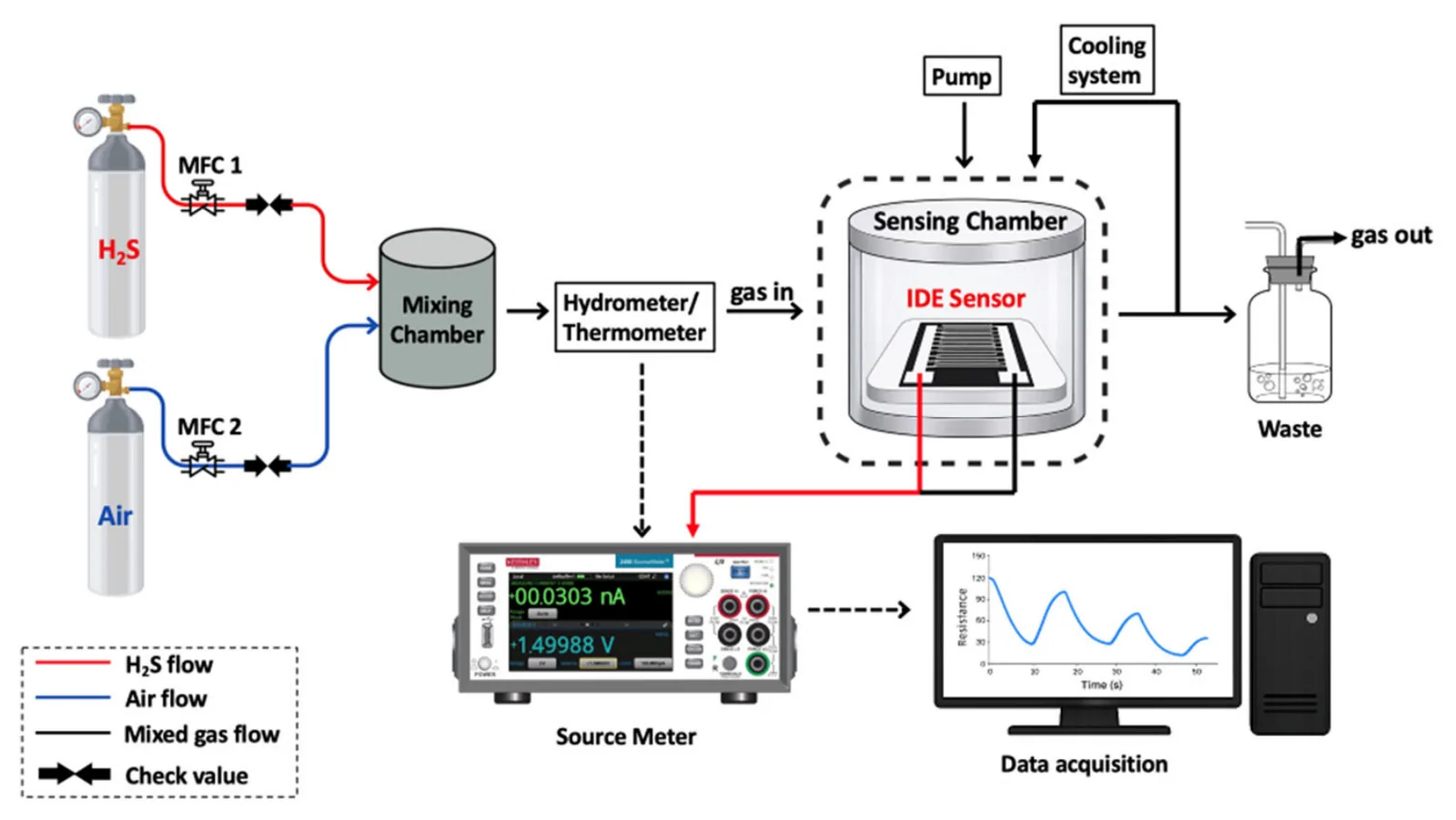
Schematic diagram of the gas sensing apparatus.

The gas-sensing evaluations were performed in a custom-designed setup featuring programmable mass flow controllers [20,35], which is shown in Scheme 6.

To continuously monitor the transient current-time (I-t) characteristics, a 1 V bias was applied to the interdigitated electrode (IDE) sensor using a Keithley 2450 SourceMeter connected via copper wires. The analytes for testing (H_2_S, NO_2_, CO, and SO_2_) were supplied from certified gas cylinders, while dry air acted as the balanced gas. Specific gas concentrations were controlled by adjusting the flow rate ratios between the dry air and the target gas based on Equation (1):

(1) $$Gas\;concentration\;\left(ppm\right)=\;\frac{Flow\;rate\;of\;analyte\;\left(sccm\right)}{\;1000\;sccm}\times 50\;ppm$$

Throughout the experiments, the total gas flow rate was strictly maintained at 1000 sccm. For instance, generating a 10 ppm gas mixture required combining 200 sccm of the target gas with 800 sccm of air. To evaluate the sensor’s gas interference and repeatability, a 10 ppm H_2_S concentration was specifically chosen. This concentration aligns with the permissible exposure limit (PEL) set by the Occupational Safety and Health Administration (OSHA), serving as a practical benchmark that allows for easy comparison with previous studies. Before initiating the tests, the sensors underwent a one-hour stabilization period in ambient air at 25 ± 0.5 °C and approximately 60% relative humidity (RH). The actual testing procedures involved exposing the sensors to analyte concentrations ranging from 1 to 10 ppm. To accurately mimic real-world operating conditions, the gas streams were pre-humidified to around 60% RH in a mixing chamber before they reached the sensor.

To ensure clear comparisons between different gas sensors, the response values of the fabricated sensors were normalized using Equation (2) [36]:

Response (%) = (R_o_ − R)/R_o_ × 100 (2)

In this equation, R and R_o_ represent the steady-state resistance values measured in the target gas and in standard air, respectively. 

The sensor’s limit of detection (LOD) is calculated using Equation (3) [37]:

LOD = 3σ/S(3)

Then, 25 successive data points in air were taken to calculate the standard deviation (σ) and S is the slope of the calibration curve.

Furthermore, the response time (tres) is defined as the duration required for the signal to reach 90% of its maximum response value following the introduction of H_2_S. Conversely, the recovery time (tresc) is the period it takes for the signal to decrease to 10% of its peak value once the environment is switched back to ambient air [38].

## 3. Results and Discussion

### 3.1. Characterization of Electroactive Monomers (BATAT, DFAT, SCEDA)

**Figure 1:**
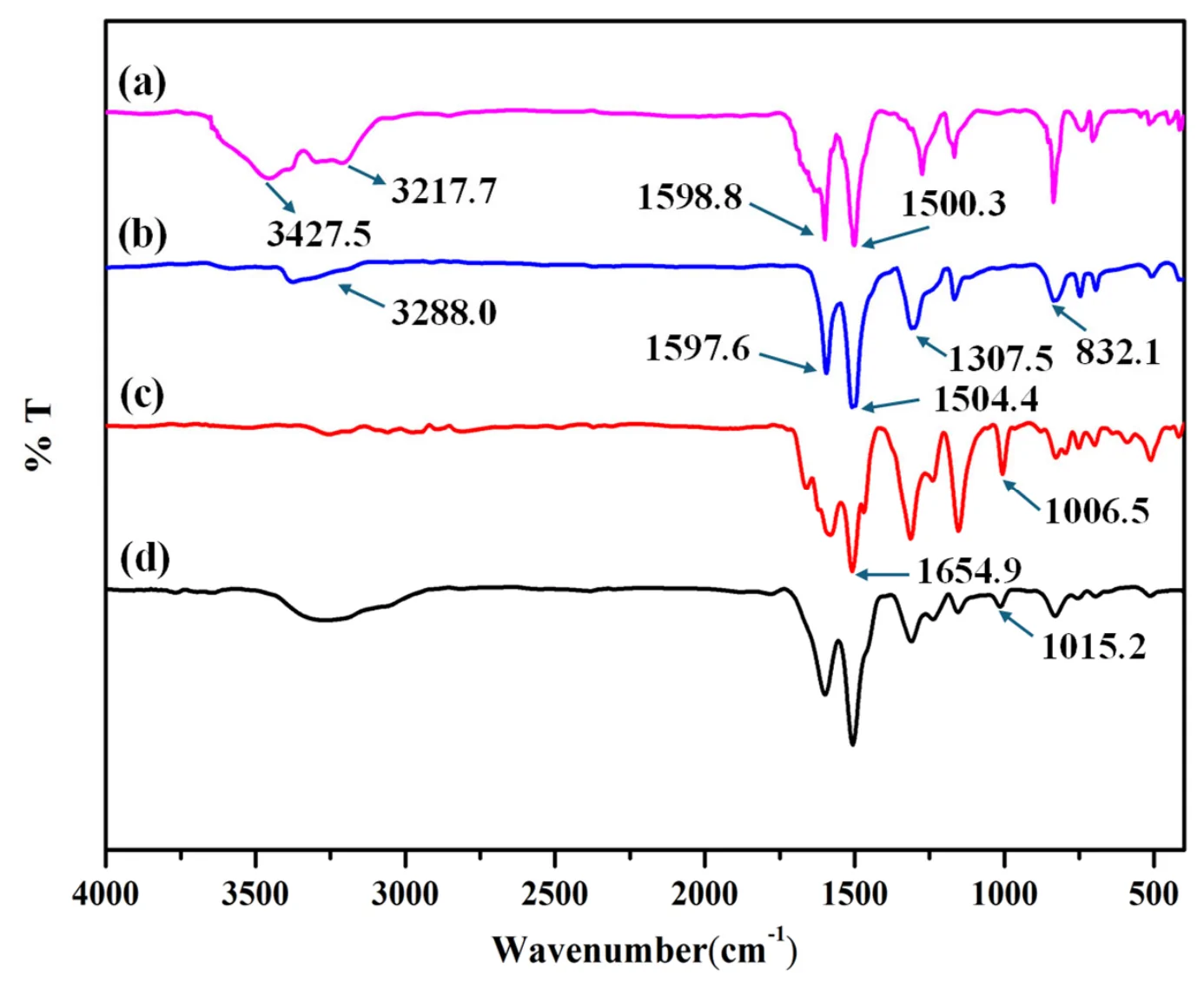
FT-IR spectra of electroactive monomers (**a**) BATAT, (**b**) UATAT, (**c**) DFAT, and (**d**) SCEDA.

**Figure 2:**
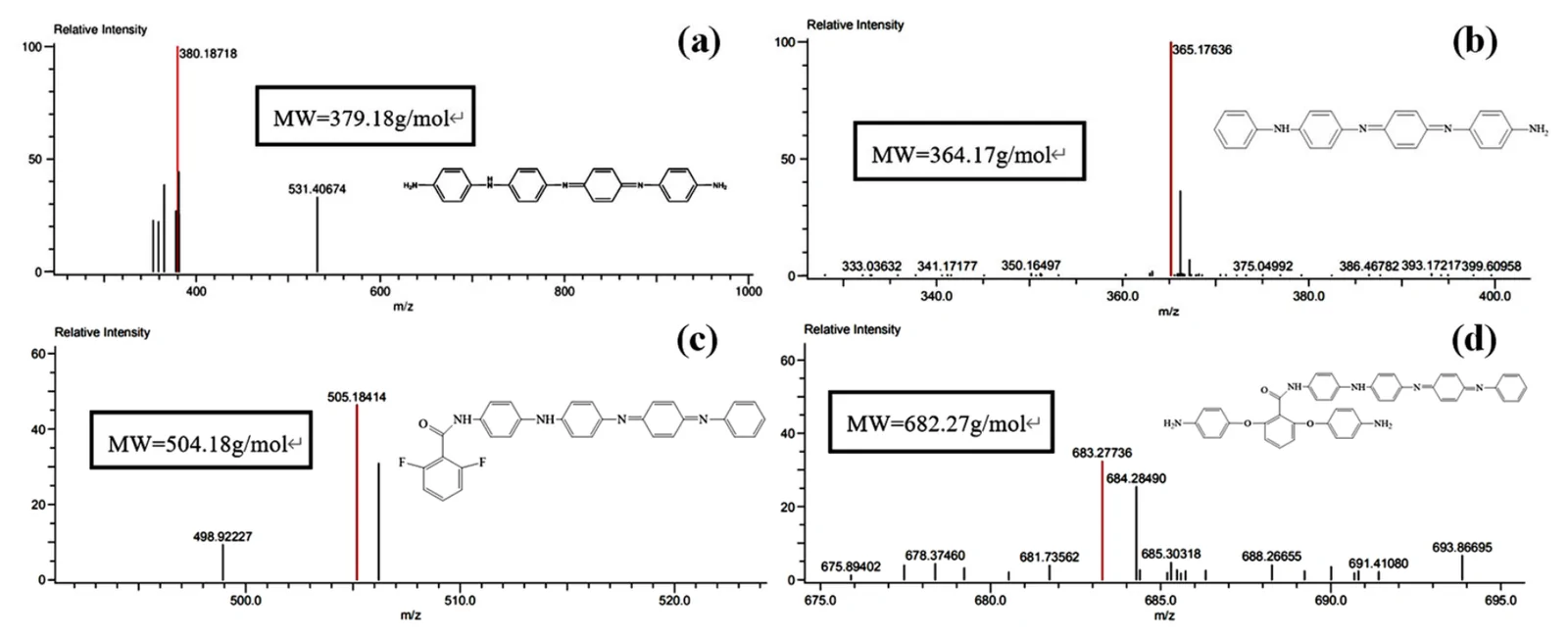
Mass spectrum of electroactive monomers (**a**) BATAT, (**b**) UATAT, (**c**) DFAT, and (**d**) SCEDA.

Figure 1 and Figure 2 present the representative FTIR and LC-MS spectra for BATAT, UATAT, DFAT, and SCEDA, with Figure 1 specifically validating the FT-IR profiles of these electroactive monomers. As depicted in Figure 1a, the distinct FTIR absorption bands located at 3427.5 and 3217.7 cm^−1^ can likely be attributed to the terminal -NH_2_ group of BATAT. Furthermore, the absorption peaks at 1598.6 and 1500.3 cm^−1^ correspond to the vibrational bands of the quinoid (Q) and benzenoid (B) rings, respectively. The structural verification of UATAT is illustrated in Figure 1b through its defining functional groups. Specifically, the out-of-plane vibrations of hydrogen atoms on the benzene ring appear at 832.1 cm^−1^, while the C–N bond absorption of the amine group is evident at 1307.5 cm^−1^. Additionally, the peaks at 1504.4 and 1597.6 cm^−1^ signify the benzenoid (N=B=N) and quinoid (N=Q=N) C=C rings, respectively, with the N-H stretching absorption peak observed at 3288.0 cm^−1^ [39]. Figure 1c confirms the functional groups of DFAT. Along with retaining the core signals of AT1, DFAT displays new characteristic peaks resulting from chemical substitution, notably a C–F stretching signal at 1006.5 cm^−1^ and a C=O absorption peak from the acyl chloride group at 1654.9 cm^−1^. Finally, Figure 1d demonstrates the verification of SCEDA. Following further chemical substitution, the C–F stretching signal previously seen in DFAT disappears, replaced by a characteristic C–O ether bond absorption peak emerging in a similar region at 1015.2 cm^−1^. Collectively, these FT-IR functional group analyses serve as preliminary evidence for the successful synthesis of both UATAT and SCEDA in this study.

As depicted in Figure 2a–d, the molecular ion peaks at 379.47, 364.17, 504.18, and 682.27 correspond to the molecular weights of BATAT, UATAT, DFAT, and SCEDA, respectively. Furthermore, analysis utilizing positive-ion mode mass spectrometry yielded mass-to-charge ratio (*m*/*z*) signals of 365.2, 380.2, 505.2, and 683.3 [M + H]^+^, respectively. Overall, these experimental findings demonstrate strong consistency with the theoretical molecular weights of BATAT, UATAT, DFAT, and SCEDA [24,34,40,41].

### 3.2. Characterization of EPAA

**Figure 3:**
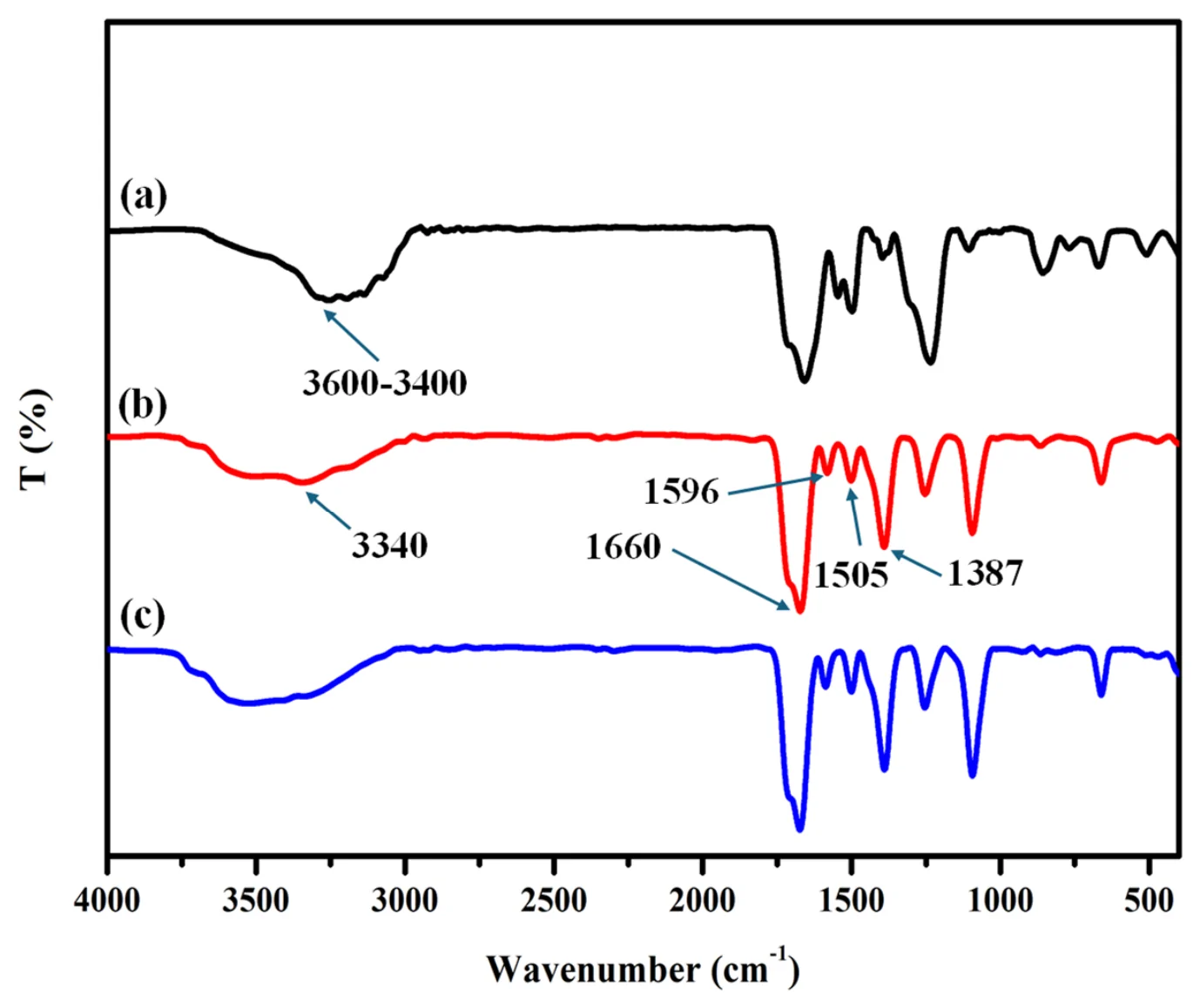
ATR-FTIR spectra of (**a**) L-EPAA, (**b**) B-EPAA, and (**c**) B-EPAA-ES.

The synthesized electroactive diamines, BATAT and SCEDA, along with the diamine ODA, were subsequently reacted with the PMDA dianhydride to produce both linear and branched electroactive poly(amic acid) (EPAA). Figure 3 presents the ATR-FTIR spectra for these materials. As illustrated in Figure 3a,b, the characteristic peaks of the prepared L-EPAA and B-EPAA observed in the 3400–3600 cm^−1^ wavenumber region correspond to the hydroxyl groups originating from the carboxylic acid of the EPAAs. Additionally, a distinct peak at 3340 cm^−1^ is assigned to N–H stretching vibrations. Two major peaks located at 1596 cm^−1^ and 1505 cm^−1^ are attributed to the quinoid and benzenoid C=C stretching modes of the aniline tetramer, respectively. The successful formation of the poly(amic acid) backbone structure is confirmed by the presence of amide C=O stretching at 1660 cm^−1^ and C–N bending at 1387 cm^−1^. Finally, as shown in Figure 3c, fabricating a porous fiber film from the branched EPAA via electrospinning (B-EPAA-ES) did not result in any alteration of its functional groups when compared to the original B-EPAA.

Weight-average molecular weight (*M_w_*), number-average molecular weight (*M_n_*), and polydispersity index (PDI) of L-EPAA and B-EPAA are shown in Table 1. As shown by the data in Table 1, B-EPAA possesses a PDI of 1.51 and, with higher-weight molecular weight, it exhibits good polymerization control without noticeable interference arising from the grafting of electroactive side-chain aniline tetramers. In contrast, although L-EPAA achieves a narrower PDI (1.25), the incorporation of aniline tetramers as main-chain repeat units introduces strong conjugated electronic effects and steric hindrance that may restrict chain propagation, thereby decreasing polymerization efficiency and resulting in an *M_w_* slightly lower than that of B-EPAA [42].

### 3.3. Electrochemical Redox Properties of EPAA

**Figure 4:**
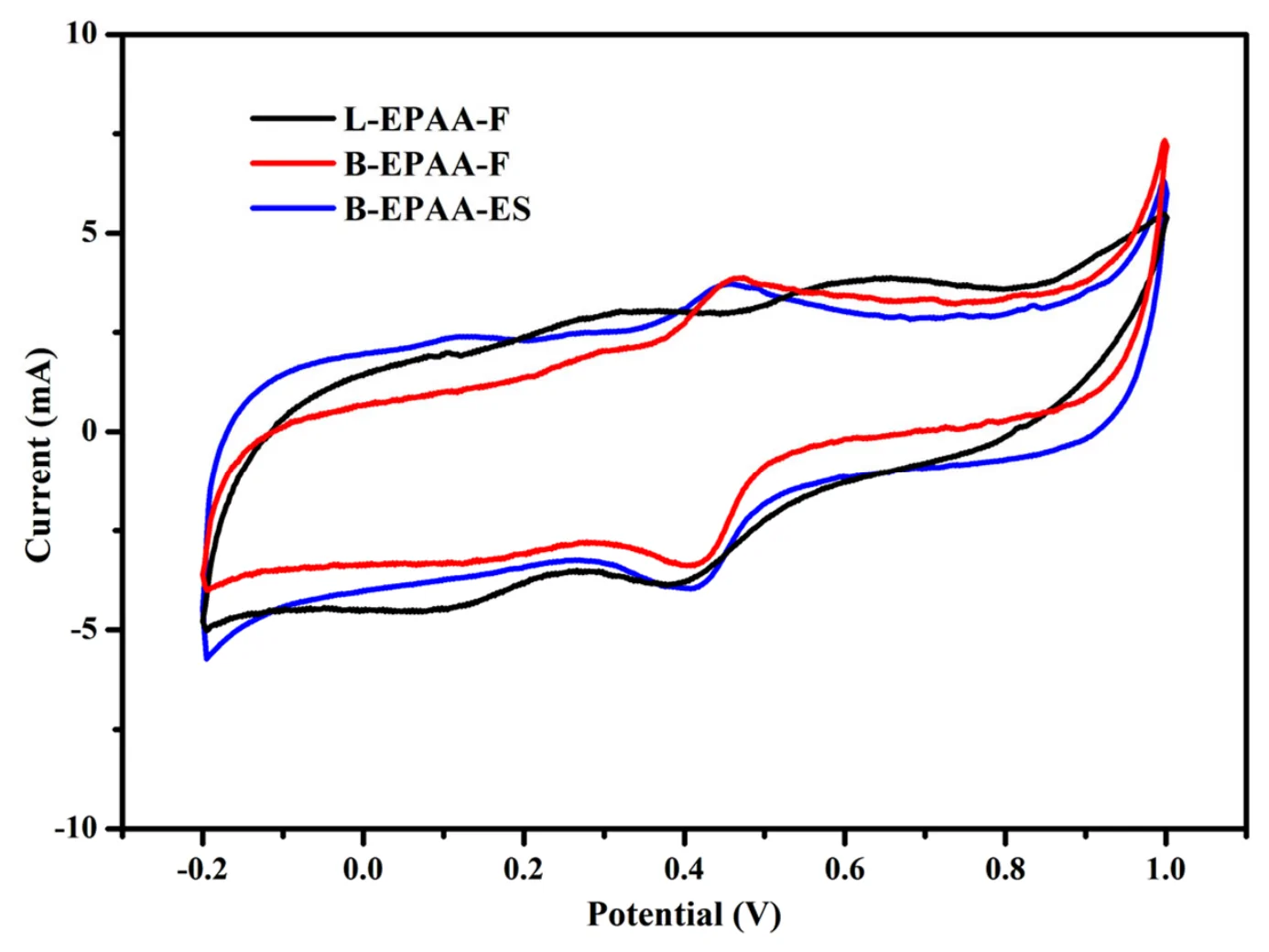
Cyclic voltammogram showing the redox capabilities of L-EPAA, B-EPAA, and B-EPAA-ES.

To evaluate their oxidation-reduction potential, all synthesized electroactive materials were tested in a highly acidic 1.0 M aqueous H_2_SO_4_ solution, operating within a potential range of −0.2 V to 1.0 V at a scan rate of 100 mV s^−1^. As shown in Figure 4, L-EPAA, B-EPAA, and B-EPAA-ES demonstrated specific redox behaviors. EPAA displayed two clear redox pairs, which correspond to the progressive oxidation stages of the aniline tetramer. In the beginning, all nitrogen atoms are in the fully reduced leucoemeraldine state, consisting entirely of amine nitrogens. The initial oxidation phase changes half of these amines into imines to form the emeraldine state, while the subsequent oxidation phase converts the rest of the amines into imines, resulting in the pernigraniline state [43]. Furthermore, fabricating the branched EPAA into a porous fiber film (B-EPAA-ES) via electrospinning does not compromise its inherent redox properties.

### 3.4. Conductivity Test of EPAA by Four-Probe

The initial electrical conductivities of the EPAA thin films were measured using a four-probe configuration. Following this baseline assessment, the films were subjected to hydrogen sulfide (H_2_S) gas, which acted as a dopant, and the subsequent rise in conductivity was recorded. To initiate the dedoping process, the samples were dried to eliminate the H_2_S, and their conductivities were evaluated again. A second exposure to H_2_S (redoping) was then conducted alongside a fourth conductivity measurement to evaluate how reversible and repeatable the doping mechanism is (Table 2). The introduction of H_2_S increased the conductivity by approximately 50 times compared to the pristine, undoped state, highlighting the potent p-type doping capability of the sulfur compounds. Upon drying and removing the H_2_S gas, the conductivity levels dropped back close to the initial baseline, verifying the highly reversible nature of this doping. The re-introduction of H_2_S successfully spiked the conductivities once more, proving the material’s strong doping endurance and its suitability for multiple, degradation-free gas sensing applications.

### 3.5. Electrospinning of B-EPAA

#### 3.5.1. Effect of Applied Voltage

**Figure 5:**
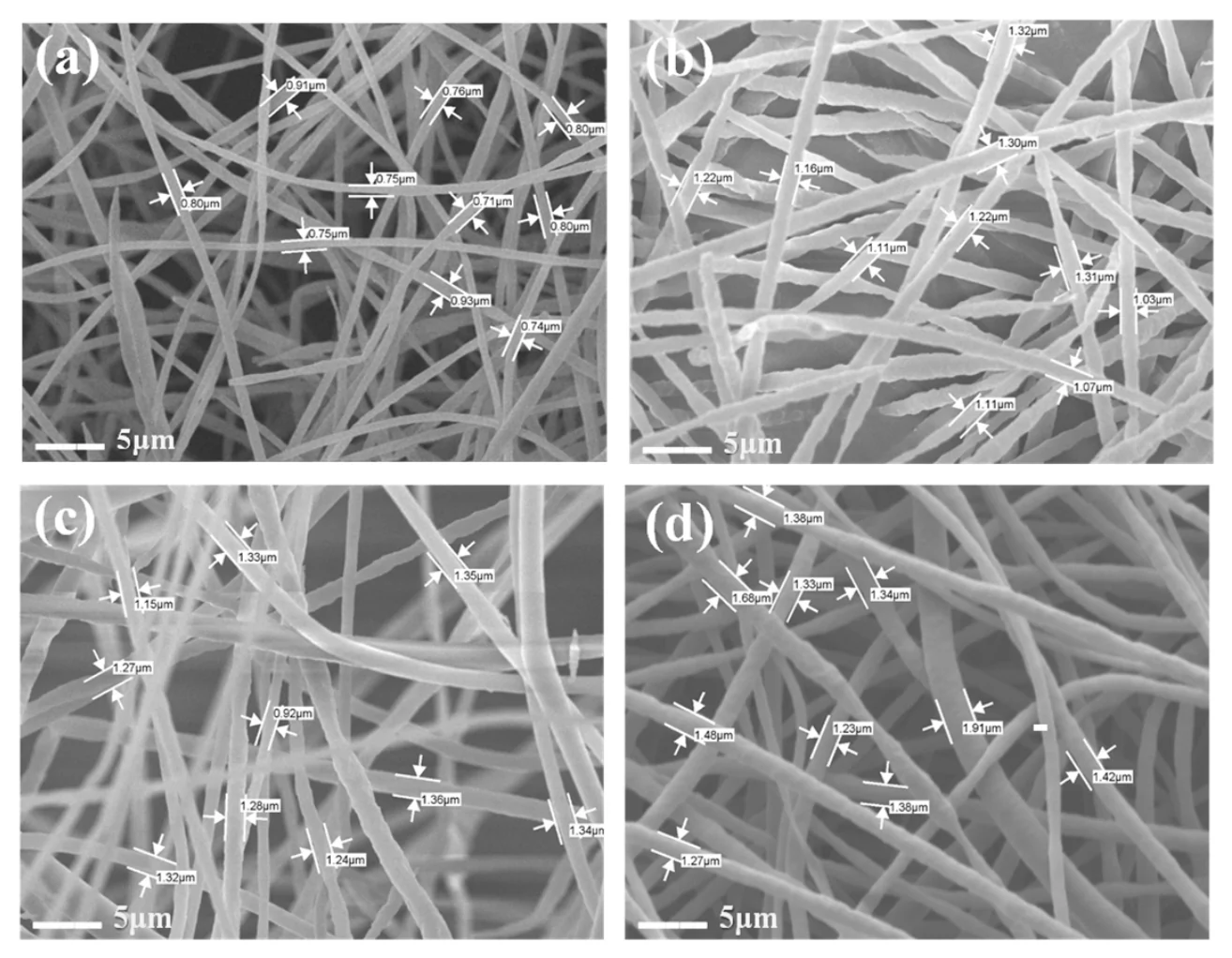
SEM images of B-EPAA-ES with different electrospinning parameters of (**a**) B-EPAA-ES-1, (**b**) B-EPAA-ES-2, (**c**) B-EPAA-ES-3, and (**d**) B-EPAA-ES-4.

The parameter of applied voltage (*V*) was a critical influence on the morphology of electrospun B-EPAA fiber mats. In the viscosity of about 1500 cP B-EPAA electrospinning process, a series of tests were carried out where the parameters *Q* = 0.01 mL min^−1^ and *H* = 10 cm were held fixed. Next, the electric field effect was examined. In this regard, we experimentally showed that the fibers produced by low voltage (13 kV) were thicker than those produced by high voltage (15 kV), with an increase of approximately 280 nm, as shown in Table 3 and Figure 5a,b.

#### 3.5.2. Effect of Nozzle-to-Collector Distance

The nozzle-to-collector distance was another important parameter to affect the configuration of the electrospun B-EPAA fibrous mats. By varying *H* at fixed *V* and *Q*, the effect of *H* on the fiber dimensions was considered. To allow for solvent evaporation during jet whipping, *H* should be sufficiently greater than the jet length; otherwise, the liquid jet touches the collector or wet fibers are obtained. In addition, the jet diameters were observed to decrease with increasing distance [44]. Shown individually in Figure 5 are typical SEM images measured in tests of about 1500 cP viscosity B-EPAA solution with *V* = 13 kV and *Q* = 0.01 mL min^−1^ for nozzle-to-collector distances of 6 and 10 cm (Figure 5b,c). The results indicated that closer needle spacing led to a shorter stretching time, resulting in an increase in fiber diameter of about 70 nm, as shown in Table 3.

#### 3.5.3. Effect of Feeding Rate

At a fixed applied voltage and nozzle-to-collector distance, jet electrospinning can be carried out by controlling Q over a specific range. A very large Q causes the drop rate of the solution to be unstable during the development of a Taylor cone (i.e., a cone emitting a jet of charged particles as observed above a threshold voltage in electrospinning, electrospraying, and hydrodynamic spraying processes). The variation in the morphologies of the B-EPAA mat fibers with an increase in Q (from 0.01 to 0.03 mL min^−1^) is shown in Figure 5c,d. Faster flow rates resulted in a larger diameter, increasing the fiber diameter by about 230 nm, as shown in Table 3.

In summary, under the conditions of 13 kV applied voltage, 0.03 mL/min feeding rate, and nozzle-to-collector distances of 6 cm, B-EPAA fibrous mats (B-EPAA-ES-4) with relatively thick fiber diameters can be obtained.

### 3.6. Pore Structure Characterization via the BET Analyses

**Figure 6:**
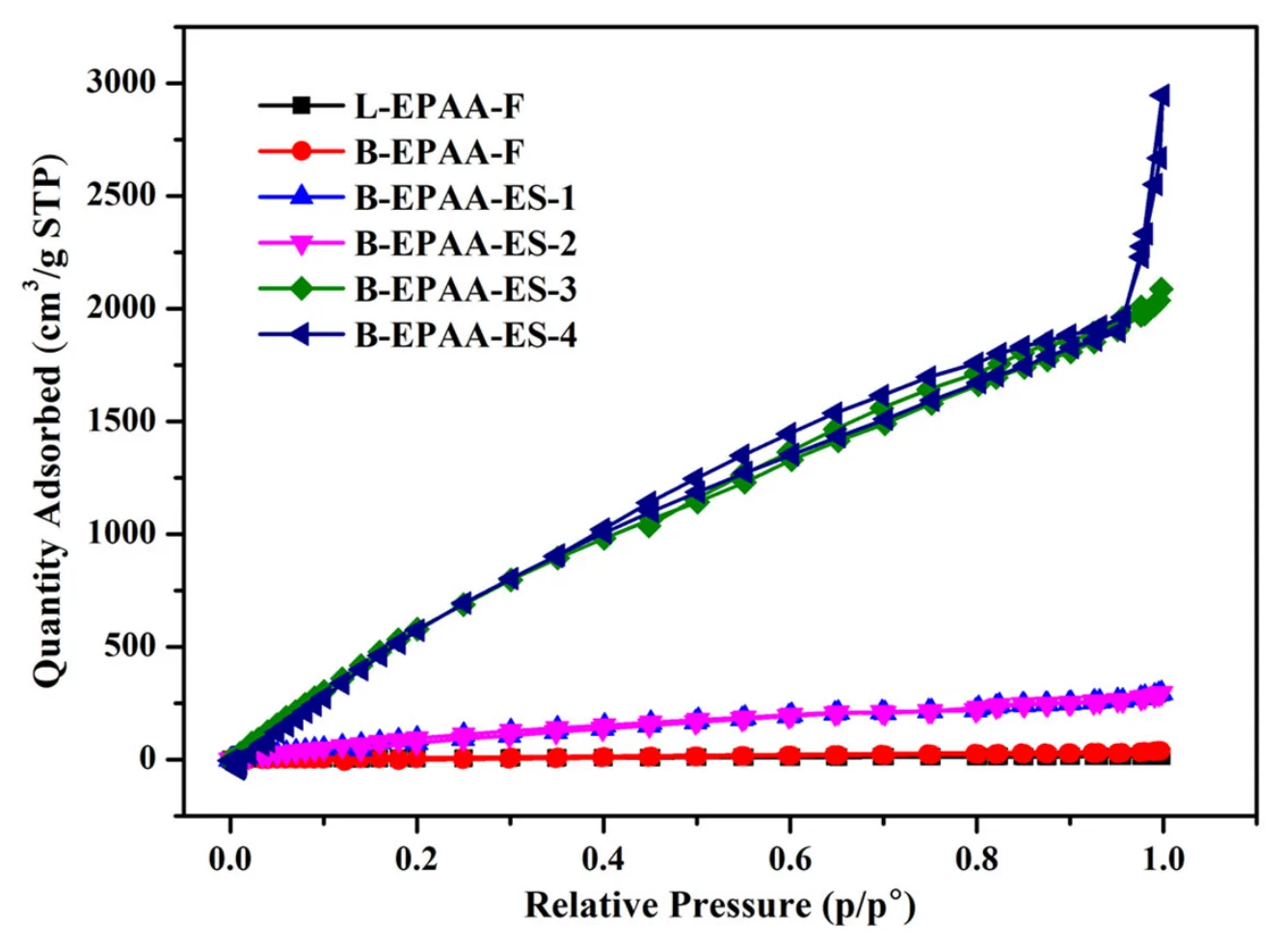
Adsorption isotherms of L-EPAA-F, B-EPAA-F, and B-EPAA-ES samples prepared under different electrospinning parameters.

This study employed N_2_ adsorption–desorption analysis at 77.35 K to investigate the specific surface area, pore volume, and average pore diameter of the samples. The equilibrium adsorption data were fitted to the Langmuir and Freundlich isotherm models using linear regression analysis. The linear region was select from 7.78 × 10^−4^ to 9.97 × 10^−1^. Table 4 provides the BET surface area values for L-EPAA-F and B-EPAA-F, which were determined to be 32 and 39 m^2^/g, respectively. Although L-EPAA-F and B-EPAA-F have similar chemical compositions, the steric hindrance effect caused by the side chains makes it more difficult for B-EPAA-F molecules to pack tightly together, which instead facilitates the formation of pores and the increase in specific surface area [45,46]. As shown in Figure 6, the adsorption/desorption curves of B-EPAA-ES revealed a Type IV isotherm with an H3 hysteresis loop. This characteristic signifies the porous structure of B-EPAA-ES [47]. The results presented in Table 4 show that B-EPAA-ES-4, with the thickest fiber diameter, which has the largest BET surface area that was measuring 5803 m^2^/g, was substantially larger than that of B-EPAA-F. Specifically, the surface area of B-EPAA-ES-4 is approximately 148 times greater than that of B-EPAA-F. The porous structure of B-EPAA-ES-4 provides additional active sites for interaction with H^+^ molecules dissociated from H_2_S.

### 3.7. Gas-Sensing Performance of L-EPAA-F, B-EPAA-F, and B-EPAA-ES Coated IDEs

#### 3.7.1. Responsivity

Detecting low levels of H2S is essential, given that the human olfactory threshold for H_2_S gas is 1.5 ppm. Therefore, we established 1 ppm as our baseline detection limit, representing the highest concentration before human detection, and evaluated sensor performance up to 10 ppm.

**Figure 7:**
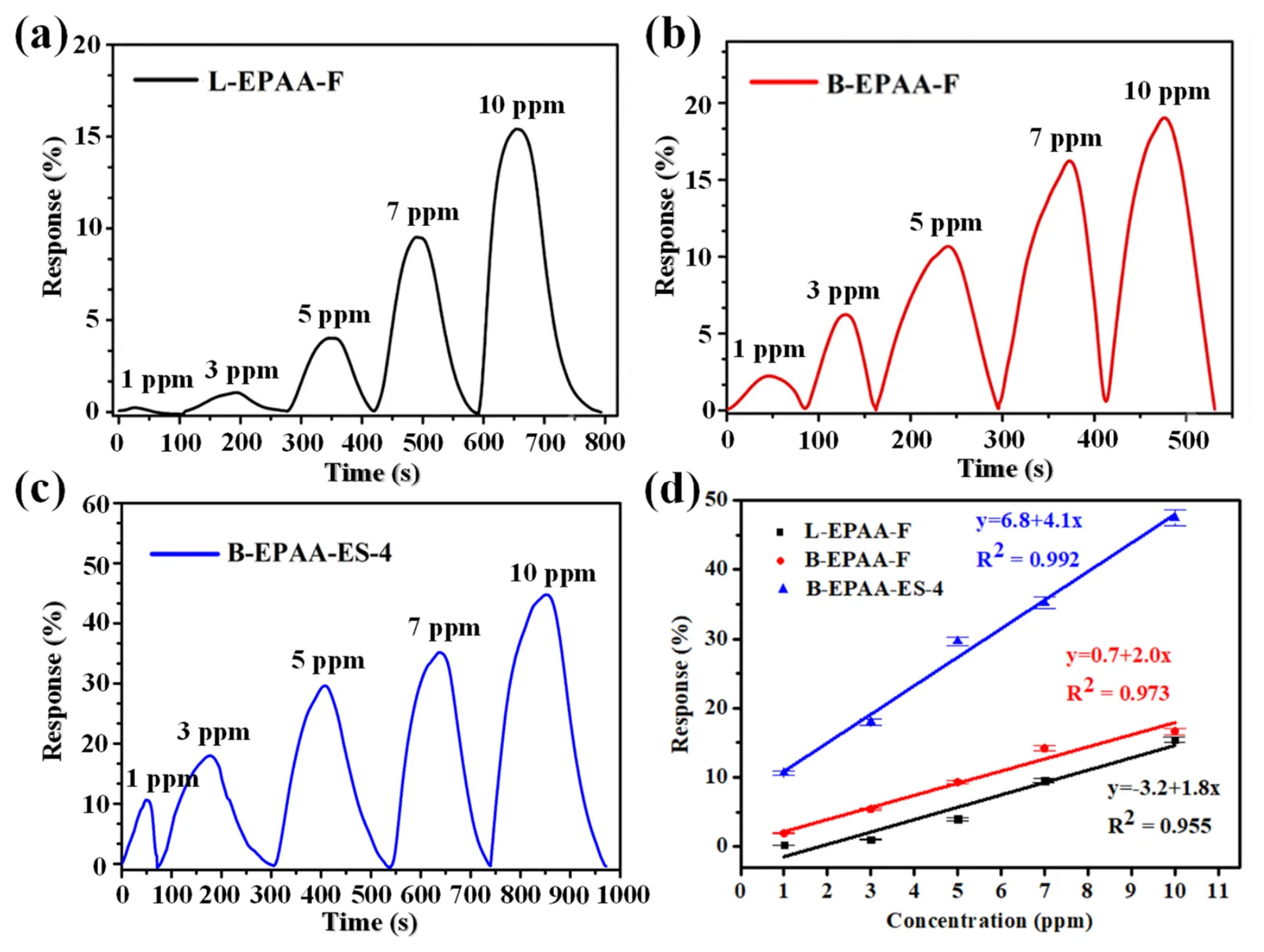
Response transient of the fabricated gas sensors to increasing concentrations of H_2_S, ranging from 1 to 10 ppm. The sensors were constructed by coating IDEs with (**a**) L-EPAA-F, (**b**) B-EPAA-F, and (**c**) B-EPAA-ES-4. (**d**) Linear calibration curves of the said sensors exposed to increasing concentrations of H_2_S.

In this study, EPAA-based sensors were utilized to detect H_2_S in an air environment. We systematically investigated and compared the sensitivity, repeatability, stability and gas interference of three different EPAA-coated IDEs. Figure 7a–c display the dynamic response curves of the L-EPAA-F, B-EPAA-F, and B-EPAA-ES-4 sensors when exposed to varying H_2_S gas concentrations of 1, 3, 5, 7, and 10 ppm. As depicted in Figure 7a, the sensor coated with L-EPAA-F exhibited response values of 0.2%, 1.1%, 4.0%, 9.5%, and 15.4% corresponding to the respective gas concentrations. In comparison, the B-EPAA-F-coated sensor yielded higher response values of 2.0%, 5.5%, 9.3%, 14.2%, and 16.7% over the same 1 to 10 ppm H_2_S concentration range, as shown in Figure 7b. Notably, at a 10 ppm H2S concentration, the B-EPAA-F sensor’s response was 1.08 times greater than that of the L-EPAA-F sensor. This enhanced performance is due to the π–π interactions created by the B-EPAA side chains, in which the aniline tetramer offers active sites for H_2_S adsorption via hydrogen bonding [48]. Consequently, B-EPAA-F demonstrates a better sensing response to H_2_S gas. The corresponding calibration curves (Figure 7d) follow the linear fits y = 1.8x + 3.2 (R^2^ = 0.955) for L-EPAA-F and y = 2.0x + 0.7 (R^2^ = 0.973) for B-EPAA-F, demonstrating reliable quantification over the tested range compared to L-EPAA-F. Additionally, the LOD of L-EPAA-F and B-EPAA-F was calculated as 0.86 ppm and 1.92 ppm using Equation (3), respectively.

As illustrated in Figure 7c, the B-EPAA-ES-4 sensor achieves remarkable responses of 10.6%, 18.0%, 29.6%, 35.2%, and 47.4% towards 1 to 10 ppm H_2_S, outperforming both EPAA-F sensors. Notably, the B-EPAA-ES-4 sensor exhibits the highest response among the three sensors, which is approximately 30% greater than those of the EPAA-F sensors. This enhanced performance can be attributed to the exceptionally high BET surface area of B-EPAA-ES-4 (5803 m^2^/g), which facilitates stronger gas adsorption. Furthermore, the corresponding calibration curve (Figure 7d) across different concentrations yields an R^2^ value of 0.992, indicating excellent sensor linearity, with a LOD of 0.39 ppm. 

#### 3.7.2. Effect of Humidity

**Figure 8:**
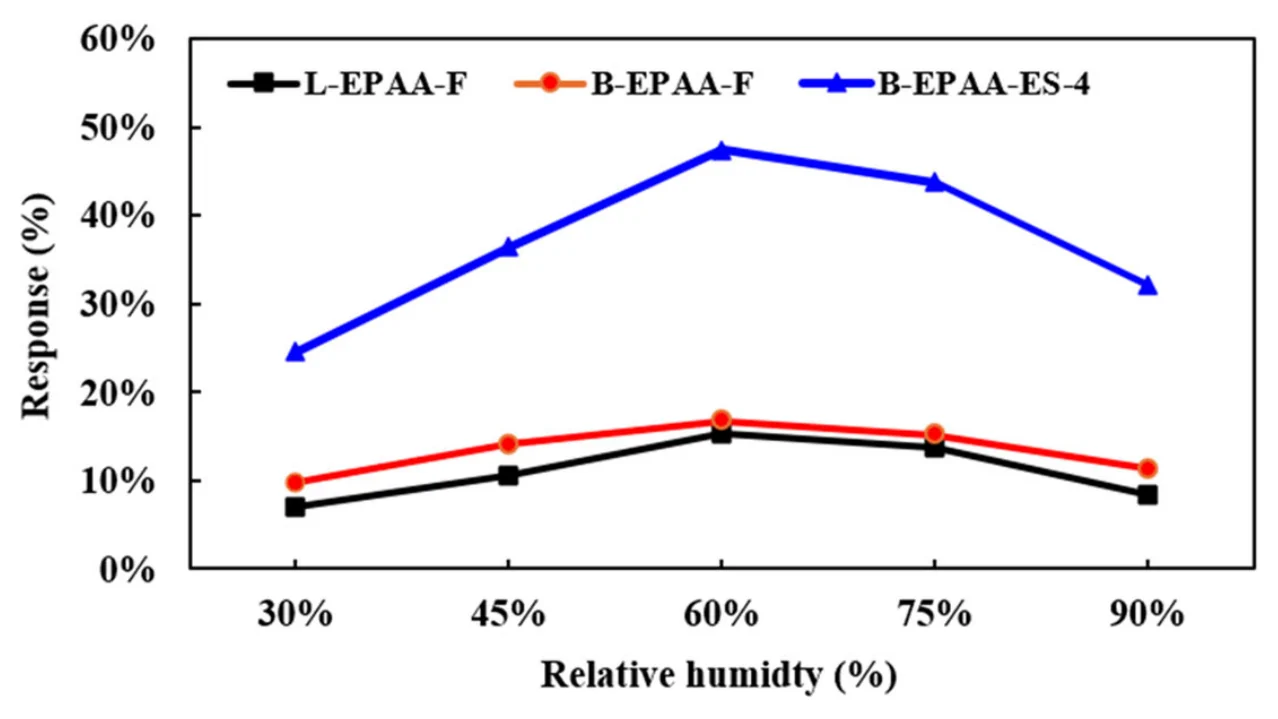
Comparison response of the L-EPAA-F-coated, B-EPAA-F-coated, and B-EPAA-ES-4-coated IDE sensors exposed to 10 ppm H_2_S with 30%, 45%, 60%, 75%, and 90% RH under room temperature (25 ± 0.5 °C).

Humidity plays a complex role in modulating chemiresistive sensors by affecting water adsorption, proton availability, interfacial reaction kinetics, and electrode stability under extreme conditions [36]. Within EPAA films, absorbed water molecules promote the dissociation of H_2_S into H^+^ and HS^−^, thereby boosting the doping of aniline units. To examine the influence of humidity on toxic gas detection, we evaluated three EPAA-coated IDE sensors under 10 ppm H_2_S across a relative humidity (RH) range of 30% to 90% (Figure 8). All three sensors exhibited highest responses at 60% RH; however, their sensitivity toward H_2_S began to decline when RH exceeded 75%. According to previous literature [49], the conductivity of PANI sensors is strongly influenced by chamber humidity. Because polar water molecules can act as a weak acid, they dope PANI and consequently increase its baseline conductivity. As a result, higher RH leads to a reduced sensitivity of the prepared sensors toward H_2_S. At elevated humidity levels (e.g., 80% RH), PANI molecules become heavily doped, leaving no additional sites for further doping; consequently, H_2_S molecules interact primarily with H_2_O rather than PANI [50]. The EPAA sensors in this study, which incorporate aniline oligomers within their structural framework, exhibit sensing behaviors consistent with these literature findings.

#### 3.7.3. Repeatability

**Figure 9:**
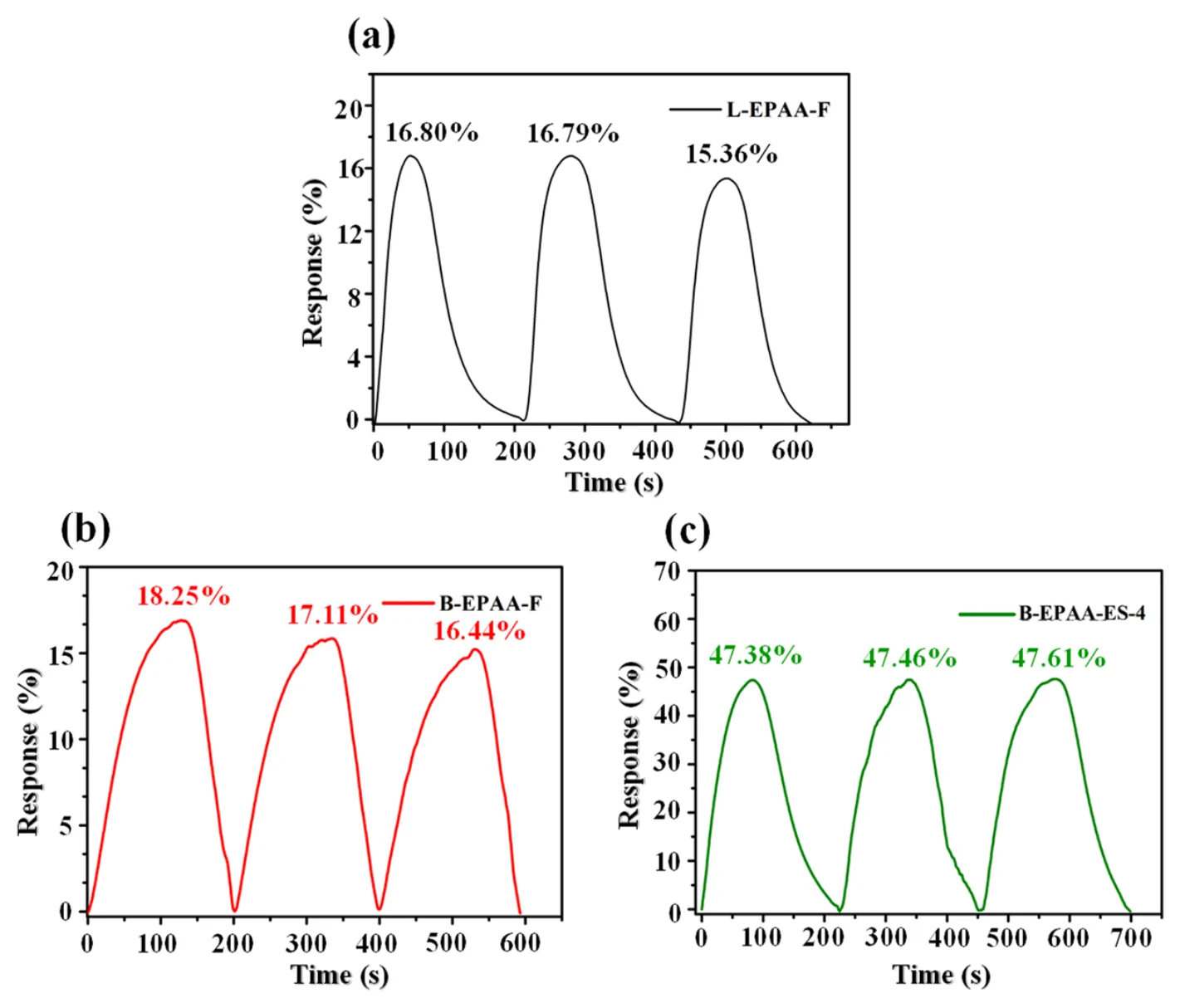
Repeatability of the fabricated gas sensors made from (**a**) L-EPAA-F, (**b**) B-EPAA-F, and (**c**) B-EPAA-ES-4 when exposed to 10 ppm H_2_S.

To assess the reproducibility of the EPAA-based sensors, repeatability tests were conducted by exposing each film (L-EPAA-F, B-EPAA-F, and B-EPAA-ES-4) to three consecutive exposure–recovery cycles using 10 ppm H_2_S gas. The average response values were recorded as 16.3% for L-EPAA-F, 17.3% for B-EPAA-F, and 47.5% for B-EPAA-ES-4. The coefficients of variation (CVs) were approximately 4.14%, 4.33%, and 0.20%, respectively. The sensors coated with L-EPAA-F and B-EPAA-F (Figure 9a,b) exhibited a slight downward trend in their response after the second cycle, and a more noticeable decline was observed in the third test. In contrast, the B-EPAA-ES-4-coated sensor maintained consistent response levels throughout repeated use, showing no significant signal degradation or baseline drift, as shown in Figure 9c. This stability is attributed to its highly uniform and continuous fiber structure, which facilitates consistent gas adsorption. Furthermore, the electrospun material not only increases the specific surface area but also encourages the orderly arrangement of side-chain aniline tetramers on the fiber surface, significantly enhancing the reproducibility of the detected conductivity changes [51,52].

#### 3.7.4. Stability

**Figure 10:**
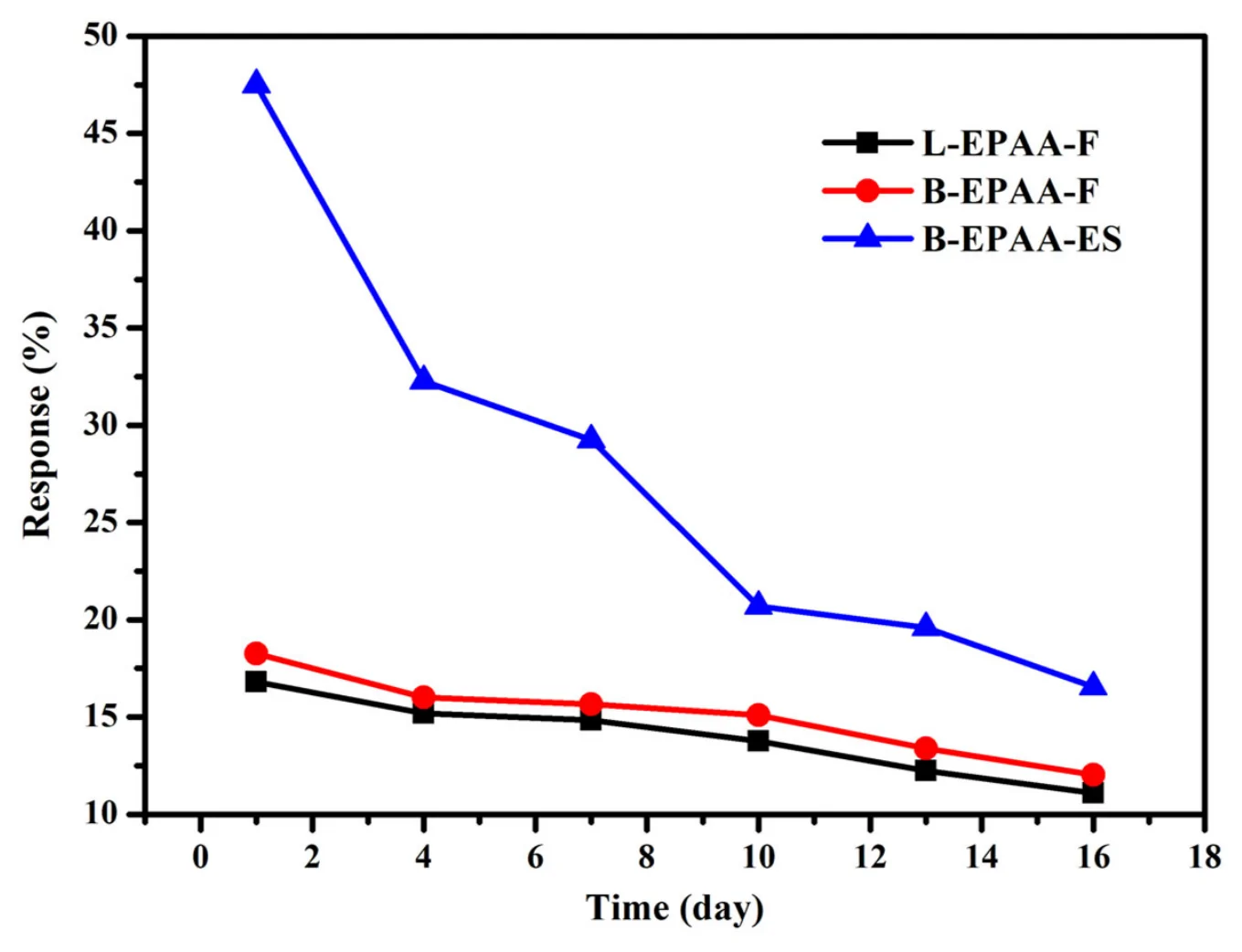
Stability of L-EPAA-F, B-EPAA-F, and B-EPAA-ES-4 sensors upon exposure to H_2_S gas (at 10 ppm) at 60% RH for 16 days.

The long-term stability of sensors based on L-EPAA-F, B-EPAA-F, and B-EPAA-ES-4 was evaluated under 10 ppm H_2_S exposure for 16 consecutive days at room temperature. The responses of the three sensors were measured at three-day intervals, and the results are presented in Figure 10. Over time, the response of all sensors gradually decreased. After 16 days, the response values of the L-EPAA-F and B-EPAA-F sensors dropped by approximately 34%, whereas the B-EPAA-ES-4 sensor exhibited a much more pronounced reduction of around 65%. The significant decline in B-EPAA-ES-4 is presumably due to its high specific surface area, which facilitates moisture absorption and subsequent hydrolysis of the amide and carboxylic acid groups in the material, thus destroying the conductive structure [53].

#### 3.7.5. Gas Interference

**Figure 11:**
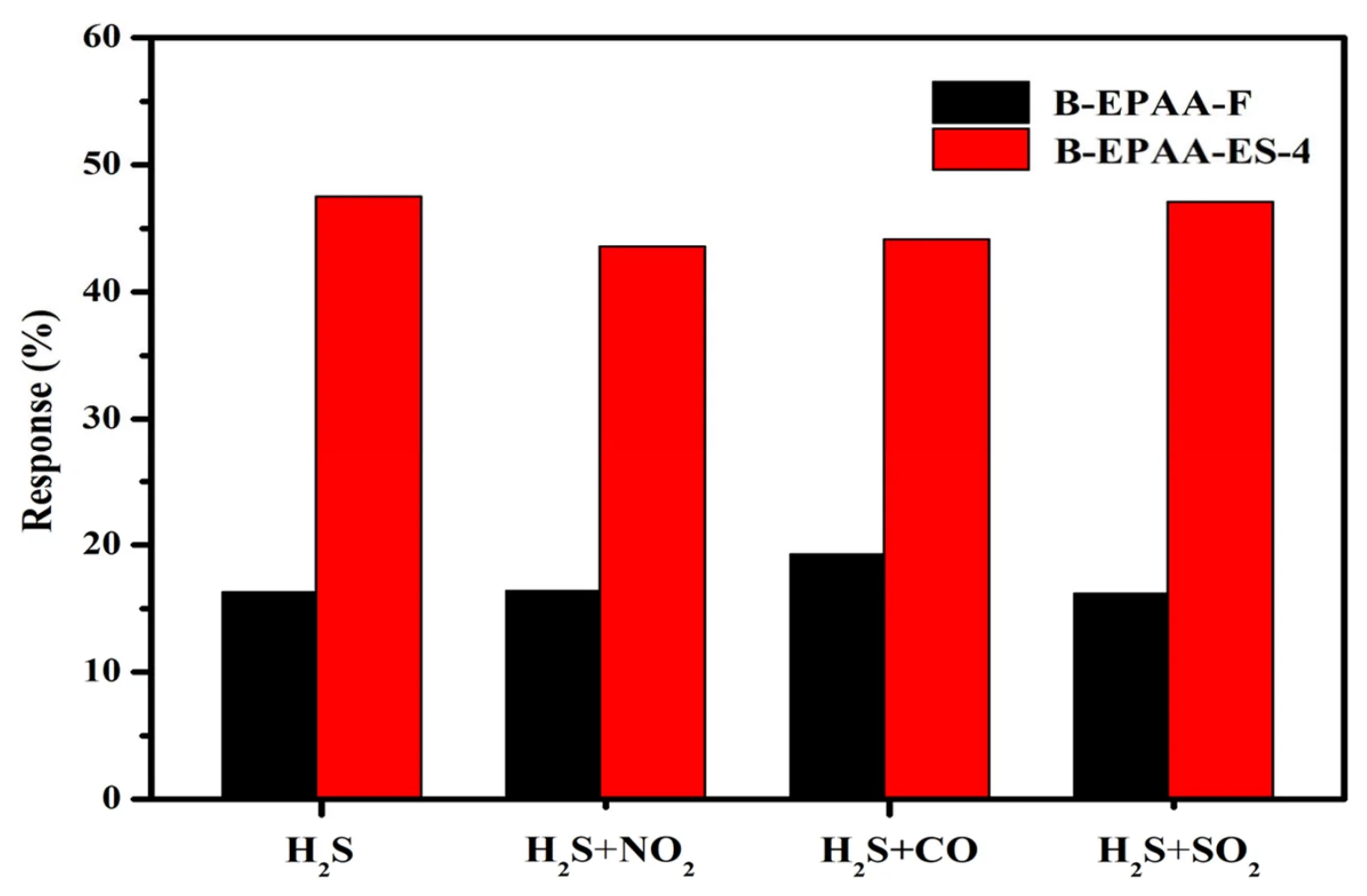
Responses (%) to simple H_2_S and different mixture gases (10 ppm H_2_S, 10 ppm H_2_S + 10 ppm NO_2_, 10 ppm H_2_S + 10 ppm CO, and 10 ppm H_2_S + 10 ppm SO_2_) of B-EPAA-F- and B-EPAA-ES-4-based gas sensors.

A gas mixing experiment was conducted to study the affection of the sensor at the 10 ppm H_2_S environment with the same concentration of other gases. The experiment was divided into 10 ppm H_2_S, 10 ppm H_2_S mixed with 10 ppm NO_2_, 10 ppm H_2_S mixed with 10 ppm CO, and 10 ppm H_2_S mixed with 10 ppm SO_2_, respectively. These tests were carried out at room temperature (25 ± 0.5 °C), under air and 60% RH. Figure 11 shows the response of two gas sensors to simple H_2_S and different mixed gases. The B-EPAA-ES-4 sensor exhibits higher response than the B-EPAA-F sensor in all tested gases. Initially, the response of B-EPAA-F and B-EPAA-ES-4 gas sensors to 10 ppm of H_2_S was 16.3% and 47.5%, respectively. The B-EPAA-ES-4 sensor maintained consistent performance despite the presence of other gases. This result can be concluded that the B-EPAA-ES-4 sensor is able to detect H_2_S even in a mixture of interfering gases and is not interfered with by other gases.

#### 3.7.6. Response/Recovery Time

**Figure 12:**
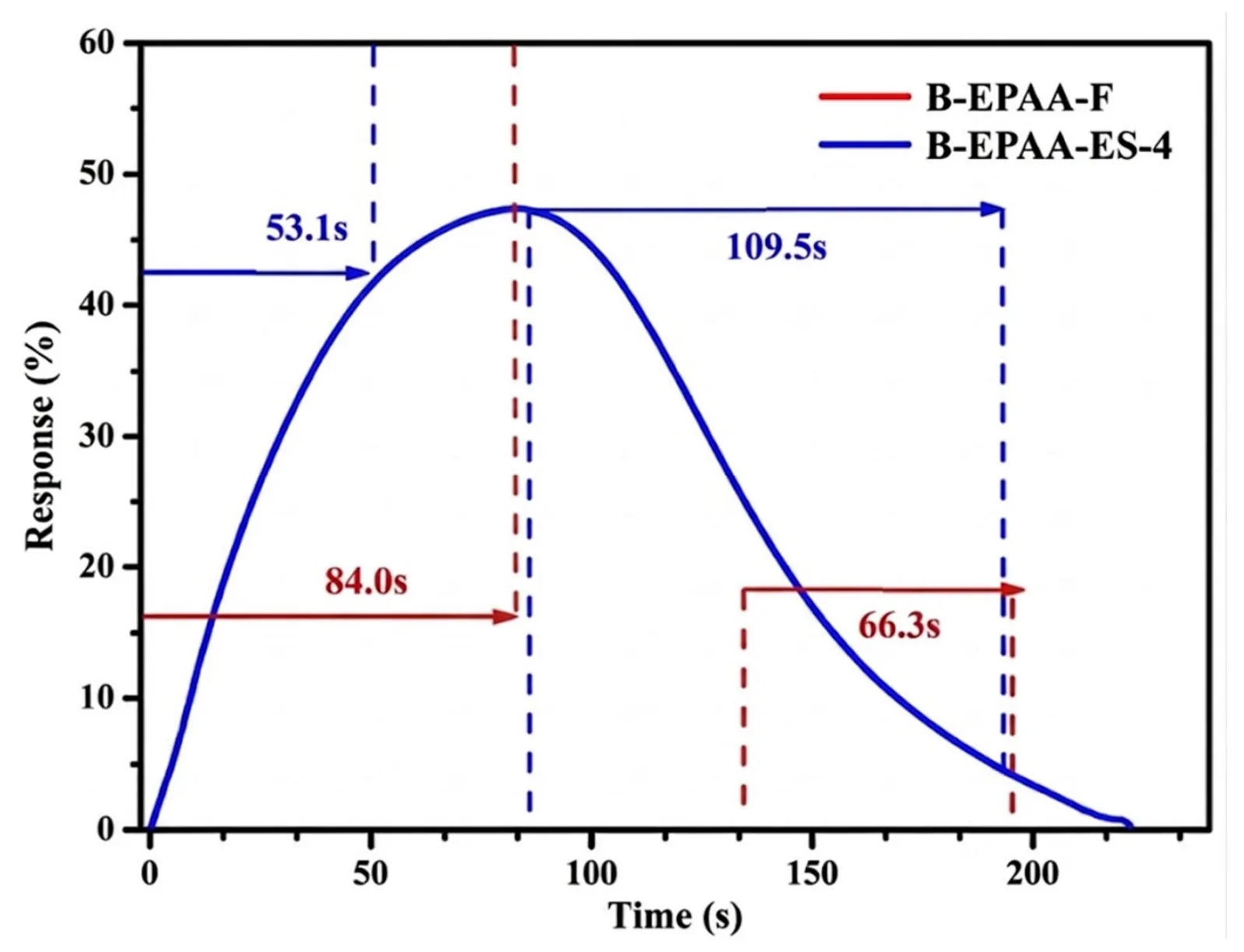
Response-recovery curve of B-EPAA-F and B-EPAA-ES-4 exposed to 10 ppm H_2_S under standard room conditions (25 ± 0.5 °C) and 60% RH.

Response–recovery time is a critical parameter in evaluating gas-sensing properties. Upon exposure to target gas, adsorption and desorption occur concurrently, meaning that the response–recovery duration is determined solely by the combined adsorption/desorption rate. A comparative analysis of response signals under 10 ppm H_2_ S gas revealed that the IDE sensor coated with B-EPAA-ES-4 exhibited a significantly stronger response than B-EPAA-F, as illustrated in Figure 12. Specifically, the response of B-EPAA-ES-4 reached 47.4%, compared to only 16.7% for B-EPAA-F. Furthermore, the B-EPAA-ES-4-coated sample demonstrated a faster response time of 53.1 s, compared to 84.0 s for B-EPAA-F. This enhanced reactivity is directly attributed to the expanded surface area provided by the porous architecture of B-EPAA-ES-4.

Table 5 summarizes related works by various researchers who utilized electroactive (conductive) polymers as sensors for H_2_S detection. The electroactive (conductive) polymer-based H_2_S sensors have different structures and morphologies. The EPAA sensor prepared in this study exhibited better response to H_2_S gas at 25 °C and 60% relative humidity. In our previous study [30], we assessed a linear-type EPAA containing an oligoaniline trimer in its main chain, utilizing electrospinning to fabricate films, beads, beads-fibers, and fibers, among which the fibrous morphology (porous film) demonstrated the superior sensing capability. In the present study, both linear (L-EPAA) and branched (B-EPAA) EPAA structures were synthesized, and the branched variant was subsequently processed into porous films via electrospinning. Notably, the sensing response (%) of the B-EPAA porous film prepared in this work is more than double that reported in the literature [30]. Additionally, this porous film exhibits outstanding reproducibility—evidenced by a coefficient of variation of only 0.2% across three successive test cycles—along with H_2_S response unaffected by other gases.

### 3.8. Proposed Sensing Mechanism

The gas-sensing mechanism of intrinsic conducting polymer (ICP)-based [58] sensors heavily rely on the doping/de-doping process. Similar to polyaniline (PANI), aniline oligomer-based poly(amic acid) (PAA) can undergo doping through either redox reactions or protonation. As a reducing gas, H_2_S can undergo ionization in the presence of water, as depicted in Equation (4). The resulting protons subsequently dope the PAA polymer in a reversible manner [59], as described in Equation (5).

H_2_S ⇌ HS^−^ + H^+^ (4)

PAA + H^+^ ⇌ PAAH^+^ (5)

**Figure 13:**
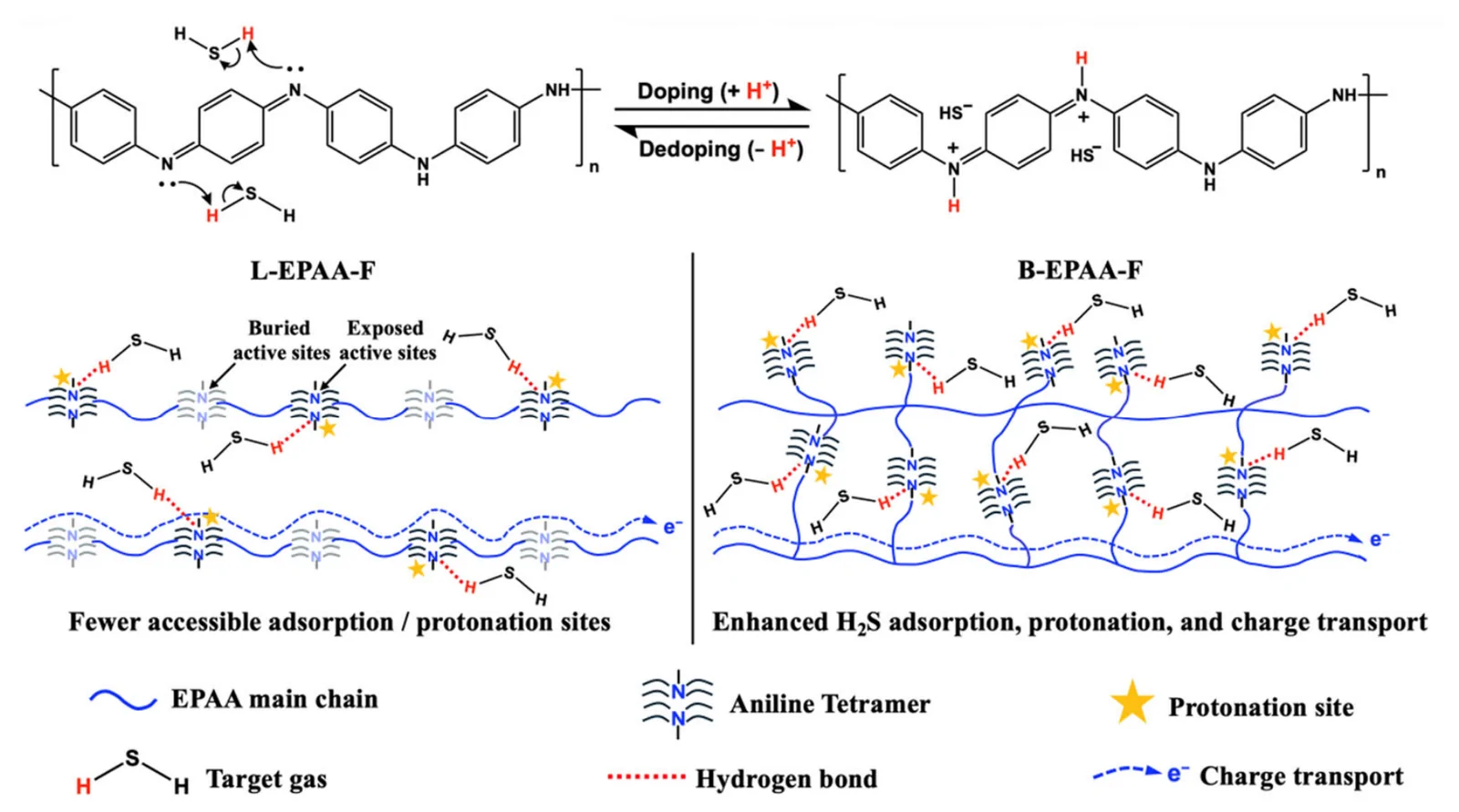
Graphical illustration of H_2_S-gas sensing for L-EPAA-F and B-EPAA-F.

When the reducing gas hydrogen sulfide (H_2_S) enters a humidified sensing environment, it dissolves in the adsorbed water layer on the material and partially dissociates into H^+^ and HS^−^ ions. These liberated protons reversibly protonate the nitrogen-containing active sites of the aniline tetramer units, which enhances mobile charge carrier density and facilitates electron hopping between neighboring conjugated segments, thereby boosting the electrical conductivity of the sensing layer. Notably, branched poly(amic acid) (B-EPAA) exhibits a superior H_2_S response compared to its linear counterpart, L-EPAA. This enhanced sensitivity is attributed to B-EPAA’s higher density of pendant aniline tetramer groups distributed along its branched polymer backbone, which provide more accessible protonation sites, shorten the hopping distance between conjugated domains, and promote the formation of continuous conductive pathways, as shown in Figure 13.

**Figure 14:**
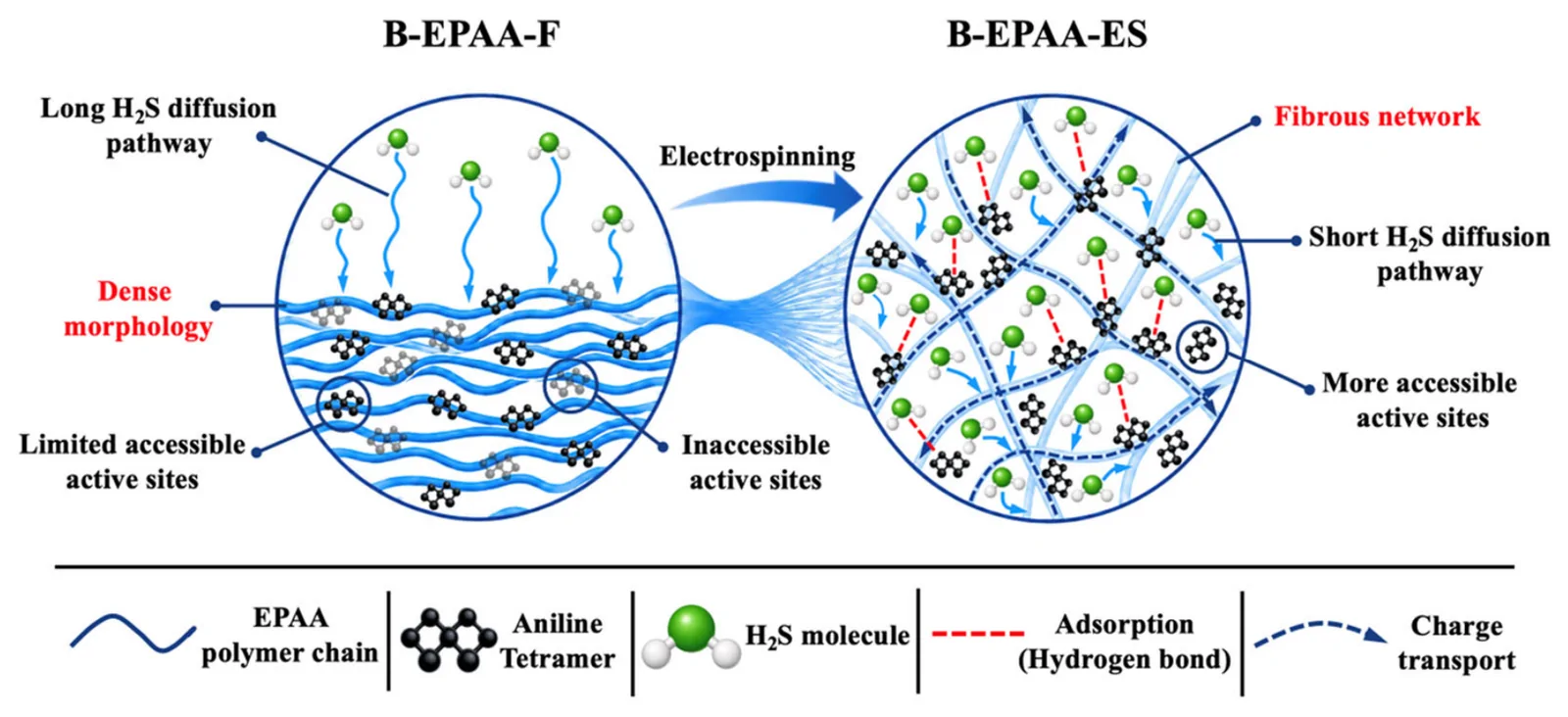
Schematic diagram for H_2_S-gas sensing mechanism by B-EPAA-F and B-EPAA-ES.

Prior to electrospinning, the dense EPAA film exhibits a limited gas-accessible surface area and longer diffusion pathways, which restrict the transport of H_2_S to internal active sites. In contrast, electrospinning transforms this dense film into an interconnected, porous nanofiber network characterized by a higher specific surface area, enhanced pore accessibility, and shortened gas diffusion paths [60,61]. Consequently, a greater number of aniline tetramer active sites are exposed to humid H_2_S, facilitating rapid protonation and a more pronounced change in conductivity, as shown in Figure 14. Thus, this porous electrospun architecture is directly responsible for the enhanced H_2_S response of B-EPAA-ES.

## 4. Conclusions

In this study, two structurally electroactive poly(amic acid)s (EPAAs)—a linear-type (L-EPAA) and a branched-type (B-EPAA)—were synthesized via condensation polymerization. This process utilized electroactive diamine monomers containing linear and pendant side-chain double-terminal aniline tetramers, and the resulting materials were subsequently characterized using ATR-FTIR and GPC. The redox capability of the EPAAs was confirmed through cyclic voltammetry, which revealed two distinct redox couples. Furthermore, four-probe conductivity measurements demonstrated the electrical tunability of the films, establishing a clear structure–property relationship that supports their observed gas-sensing behavior.

Electrospinning technology was employed to fabricate porous fiber membranes from the branched EPAA (designated as B-EPAA-ES). By applying a voltage of 13 kV, a feed rate of 0.03 mL/min, and a nozzle-to-collector distance of 6 cm, B-EPAA fibrous mats (B-EPAA-ES-4) were produced, featuring relatively thick fiber diameters and a high surface area.

During gas-sensing evaluations on interdigitated electrodes (IDEs), B-EPAA-ES-4 exhibited superior performance in detecting H_2_S gas at a concentration of 10 ppm compared to both L-EPAA and B-EPAA, achieving a 47.4% response, with a faster response time of 53.1 s. The material also displayed remarkable reproducibility, maintaining a coefficient of variation of merely 0.2% across three consecutive testing cycles. Additionally, the H_2_S response of B-EPAA-ES is not affected by other gases. Overall, B-EPAA-ES demonstrates a good balance of processability, responsivity, repeatability, and responsiveness unaffected by other gases.

## Figures and Tables

**Table 1:** Molecular weight and PDI of L-EPAA and B-EPAA.

Sample Code	*M_w_*	*M_n_*	PDI
L-EPAA	6,220,000	4,130,000	1.51
B-EPAA	5,460,000	4,380,000	1.25

**Table 2:** Conductivity comparison of L-EPAA and B-EPAA in intrinsic, doped, dedoped, and redoped forms.

Sample Code	Conductivity (S/cm)			
	Intrinsic	Doped	Dedoped	Redoped
L-EPAA	5.57 × 10^−7^	5.09 × 10^−5^	2.01 × 10^−7^	3.05 × 10^−5^
B-EPAA	3.38 × 10^−7^	5.05 × 10^−5^	2.97 × 10^−7^	2.82 × 10^−5^

**Table 3:** Fiber diameter of B-EPAA-ES with different electrospinning parameters.

Sample Code	Applied Voltage (kV)	Nozzle-to-Collector Distance (cm)	Feeding Rate (ml/min)	Fiber Diameter (nm)
B-EPAA-ES-1	15	10	0.01	900 ± 120
B-EPAA-ES-2	13	10	0.01	1190 ± 100
B-EPAA-ES-3	13	6	0.01	1260 ± 130
B-EPAA-ES-4	13	6	0.03	1490 ± 200

**Table 4:** BET analysis of L-EPAA-F, B-EPAA-F, and B-EPAA-ES with different electrospinning parameters.

Sample Code	S_BET_ (m^2^/g) ^a^	V_t_ (cm^3^/g) ^b^	D_p_ (nm) ^c^
L-EPAA-F	32	0.02	2.86
B-EPAA-F	39	0.06	5.76
B-EPAA-ES-1	233	0.31	2.50
B-EPAA-ES-2	645	0.45	2.78
B-EPAA-ES-3	4165	3.15	3.02
B-EPAA-ES-4	5803	4.13	2.84

^a^ BET surface area. ^b^ Total pore volume calculated from P/P_0_ = 0.99. ^c^ Pore diameter.

**Table 5:** Performance comparison of various electroactive (conductive) polymers-based H_2_S sensors with different structure and morphologies.

Materials	Aniline Oligomer	Structure	Morphology	Temp. (°C)	RH (%)	Conc. (ppm)	Response (%)	Ref.
PANI	—	Linear	Porous film (Nanofibers)	30	—	25	3.2	[54]
PANI-PEO	—	Linear	Porous film (Nanofibers)	25	70	10	~25	[50]
PANI/ZnO	—	Linear	Dense film	25	52	50	40.5	[55]
PANI-Rh-SnO_2_	—	Linear	Porous film (Hollow nanotubes)	25	50	50	~20	[56]
Ag NPs-PPy NTs	—	Linear	Dense film (Nanotubes)	25	—	20	30	[57]
EPAA	Trimer	Linear	Dense film	25	60	10	~15 ^a^	[30]
EPAA	Trimer	Linear	Porous film	50	60	10	~22.5 ^b^	[30]
EPAA	Tetramer	Linear	Dense film	25	60	10	15.4	This work
EPAA	Tetramer	Branch	Dense film	25	60	10	16.7	This work
EPAA	Tetramer	Branch	Porous film (Nanofibers)	25	60	10	47.4	This work

Response % = $(\frac{I-I_{0}}{I_{0}})\times 100\%$ = $(\frac{I}{I_{0}}-1)\times 100\%$; ^a^ I/I_0_ around 1.15, Response % = ~(1.15 − 1) * 100 = ~15%; ^b^ I/I_0_ around 1.225, Response % = ~(1.225 − 1) * 100 = ~22.5%.

## Data Availability

The data that support the findings of this study are available from the corresponding author, but restrictions apply to the availability of these data, which were used under license for the current study, and so are not publicly available. The data are, however, available from the authors upon reasonable request and with permission of the funding party, Ministry of Science and Technology, Taiwan, R.O.C.

## Abbreviations

The following abbreviations are used in this manuscript:

H_2_S	Hydrogen sulfide
EPAA	Electroactive poly(amic acid)
L-EPAA	Linear electroactive poly(amic acid)
B-EPAA	Branch electroactive poly(amic acid)
Ppm	parts-per-million
DLC	Discotic liquid crystal
PI	Polyimide
PLA	Polylactic acid
PUU	Polyurethane-urea
PMMA	Polymethacrylate
ODA	4,4′-Oxydianiline
DMF	N,N-Dimethyl formamide
PMDA	1,2,4,5-Pyromellitic dianhydride
NO_2_	Nitrogen dioxide
CO	Carbon monoxide
SO_2_	Sulfur dioxide
ITO	Indium tin oxide
FTIR	Fourier-transform infrared
ATR	Attenuated total reflectance
GPC	Gel permeation chromatography
SEM	Scanning electron microscopy
BET	Brunauer–Emmett–Teller
BATAT	Bilaterally amino-terminated aniline tetramer
UATAT	Unilaterally amine-terminated aniline tetramer
DFAT	Aniline tetramer-difluorobenzene monomer
SCEDA	Side chain electroactive diamine monomers
IDE	Interdigitated electrode
PEL	Permissible exposure limit
OSHA	Occupational safety and health administration
RH	Relative humidity
LOD	Limit of detection
M_w_	Weight-average molecular weight
M_n_	Number-average molecular weight
PDI	Polydispersity index
CV	Coefficient of variation
ICP	Intrinsic conducting polymer
PANI	Polyaniline
